# Attention and speech-processing related functional brain networks activated in a multi-speaker environment

**DOI:** 10.1371/journal.pone.0212754

**Published:** 2019-02-28

**Authors:** Brigitta Tóth, Dávid Farkas, Gábor Urbán, Orsolya Szalárdy, Gábor Orosz, László Hunyadi, Botond Hajdu, Annamária Kovács, Beáta Tünde Szabó, Lidia B. Shestopalova, István Winkler

**Affiliations:** 1 Institute of Cognitive Neuroscience and Psychology, Research Centre for Natural Sciences, Hungarian Academy of Sciences, Budapest, Hungary; 2 Department of Cognitive Science, Faculty of Natural Sciences, Budapest University of Technology and Economics, Budapest, Hungary; 3 Institute of Behavioural Sciences, Faculty of Medicine, Semmelweis University, Budapest, Hungary; 4 Department of Social and Educational Psychology, Eötvös Loránd University, Budapest, Hungary; 5 Department of General and Applied Linguistic, University of Debrecen, Debrecen, Hungary; 6 Department of Telecommunication and Media Informatics, Budapest University of Technology and Economics, Budapest, Hungary; 7 Faculty of Information Technology and Bionics, Pázmány Péter Catholic University, Piliscsaba, Hungary; 8 Pavlov Institute of Physiology, Russian Academy of Sciences, St. Petersburg, Russia; Baycrest Health Sciences, CANADA

## Abstract

Human listeners can focus on one speech stream out of several concurrent ones. The present study aimed to assess the whole-brain functional networks underlying a) the process of focusing attention on a single speech stream vs. dividing attention between two streams and 2) speech processing on different time-scales and depth. Two spoken narratives were presented simultaneously while listeners were instructed to a) track and memorize the contents of a speech stream and b) detect the presence of numerals or syntactic violations in the same (“focused attended condition”) or in the parallel stream (“divided attended condition”). Speech content tracking was found to be associated with stronger connectivity in lower frequency bands (delta band- 0,5–4 Hz), whereas the detection tasks were linked with networks operating in the faster alpha (8–10 Hz) and beta (13–30 Hz) bands. These results suggest that the oscillation frequencies of the dominant brain networks during speech processing may be related to the duration of the time window within which information is integrated. We also found that focusing attention on a single speaker compared to dividing attention between two concurrent speakers was predominantly associated with connections involving the frontal cortices in the delta (0.5–4 Hz), alpha (8–10 Hz), and beta bands (13–30 Hz), whereas dividing attention between two parallel speech streams was linked with stronger connectivity involving the parietal cortices in the delta and beta frequency bands. Overall, connections strengthened by focused attention may reflect control over information selection, whereas connections strengthened by divided attention may reflect the need for maintaining two streams in parallel and the related control processes necessary for performing the tasks.

## Introduction

Although in everyday life we can reliably follow one speech stream in a noisy multi-talker environment [1], understanding the brain’s machinery underlying this feat is one of the major challenges of auditory neuroscience. This is due to the fact that solving this problem involves complex functions, such as auditory scene analysis [2], speech processing on multiple timescales [3], and selective attention [4–8]. Previous neuroimaging data showed a distributed network of brain regions subserving the above functions (e.g.[9–13]) and there is also electrophysiological data on the temporal mechanisms of speech processing (for a review, see [14,15]). However, investigating the dynamic communication between brain regions (termed functional connectivity—FC) may provide a tool to join these two views describing the functioning of these anatomically distributed brain activity on several timescales [16,17]. By recording electroencephalographic (EEG) signals in a realistic multi-talker setup the goal of the present study was to assess the whole-brain functional networks underlying a) the process of focusing attention on a single speech stream to improve information extraction from this stream and 2) speech processing on different time-scales and depth.

### Dynamic aspects of brain activity during auditory attention and speech processing

Speech perception proceeds in parallel on different timescales from speech segmentation into units such as phonemes or syllables to the integration of phrases to sentences and sentences to the context ([18]. A growing body of EEG/MEG evidence suggests that analyzing neural oscillatory activity operating on different timescales provides information about a wider set of brain processes than the event-related brain potential methods ([18–20]. Neural oscillations in the delta (0.5–4 Hz), theta (4–8 Hz), and beta/gamma (30–70 Hz) frequency bands have been shown to be synchronized/entrained to different levels of the temporal structure of speech [21–26]. Specifically, gamma band oscillation shows alignment with phonemes [15,18], theta band oscillations synchronize with syllables, and delta band oscillation with intonational phrase boundaries and the speech envelope [22,24,27–32]. Because neural rhythms in these frequencies cover the temporal structure of the major speech units it has been proposed that temporal alignment might serve to align neural excitability to linguistically distinctive spectral information. Oscillations in different frequency ranges may serve functions such as neural segmentation and identification of various speech units [15,18,21,33,34].

Neural oscillations may also support higher-level functions of speech perception. Attentional modulation of the delta- and theta-band EEG signal (below ∼7 Hz) has been suggested to reflect a general mechanism of sensory information selection, which is based on temporal expectations[35–37]. Some recent studies delivering continuous quasi-naturalistic speech stimuli to listeners provided evidence suggesting that slow oscillatory phase-entrainment mechanisms may be responsible for both the selection and suppression of speech streams in the brain[4–8]. For instance, delta-theta responses in the posterior temporal lobe are enhanced for the attended but suppressed for the unattended speaker [5]. Others have found the significant but weaker speech-brain correlation for attended vs. unattended speech [7]. Horton and colleagues found that one of the ICA sources form the temporal cortices strongly encoded the envelope of attended speech at 200-ms delay while also encoding the inverse of the unattended speech’s envelope with the same delay. In summary both types of results show that the attended and unattended speech is distinguished in the temporal lobe. A recent study demonstrated that speech-brain correlation was modulated by attention only for natural but not for vocoded speech indicating that attention may affect higher level linguistic, rather than lower level sensory speech processing [8]. Concordantly in higher-order brain regions, selective representation of the attended speech was observed with no detectable tracking of ignored speech [6], a contrast to the findings in lower-level auditory cortical structures. These results suggest that selectively attending to a speaker increases the gain of the attended speech stream and/or reduce the gain of the competing stream by phase entrainment mechanisms in the delta-theta oscillation range [38].

Enhancement of alpha-band (8–13 Hz) activity is assumed to reflect the disengagement of cortical areas not involved in the given task[39,40]. With respect to speech processing, it has been shown that the brain regions involved in speech processing exhibit lower alpha power during listening to speech in a quiet environment than in a noisy one. The observed higher alpha activity when speech is presented together with maskers has been assumed to represent inhibition of the processing of noise, thereby protecting the processing of the task-relevant speech signal from interference [41,42]. Further, alpha power in auditory cortical areas was found to be higher contralateral to the side of noise delivery and lower contralateral to the side of speech delivery, which can be interpreted as indicating suppression of the unattended auditory input [43].

Although the exact role of beta-band activity in auditory attention has not yet been clarified, it was suggested that beta-band modulations may reflect control processes [44,45]. For example, tasks requiring enhanced top-down processing are accompanied by enhanced beta activity [46]. Studies on primates and cats have also shown sustained beta synchronization during stimulus expectation [9,46,47]. Further, voluntary engagement of auditory spatial attention has been associated with sustained high frequency (beta/gamma-band) fronto-parietal and temporal activity, which was predictive of overt task performance [48]. A more specific role of beta oscillation in expectation during speech processing has been recently suggested: beta band oscillations may mediate contextual semantic prediction of individual words based on the prior context [15,49].

### The functional anatomy and connectivity of auditory attention to speech

Neuroimaging studies investigating the functional anatomy of auditory attention revealed the involvement of distributed fronto-temporal (ventral stream) and fronto-parietal (dorsal stream) networks in dichotic listening [9–13]. The activation of the ventral pathway, primarily consisting of superior temporal and inferior frontal areas, has been shown to respond to the stimulus-driven allocation of attention [12,50]. In contrast, the dorsal fronto-parietal pathway (involving precentral areas, the medial prefrontal cortex, and the superior parietal gyrus) respond to voluntary attentional control, such as the resolution of interference from concurrent speech or noise signals [9,10,13,51].

The analysis of FC (measured as neural oscillatory phase synchronization) can potentially reveal the interplay between speech processing and attentional control functions [52] because oscillatory synchrony among cortical regions is assumed to facilitate neuronal communication [53–55]. So far, only two electrophysiological studies have investigated FC while listeners listened to speech in the presence of another active sound source [56,57]. Comparing FC between a dichotic listening condition delivering stimuli with high spectral overlap and a sham dichotic listening condition including stimuli with low spectral overlap resulted in an increase of alpha-band coherence between left auditory and Wernicke’s areas and decreased interhemispheric coherence between auditory areas [56]. Keitel and colleagues [57] presented speech stimuli embedded in noise. They have demonstrated that 1) speech tracking existing on several linguistically relevant timescales (timescales of phrases (0.6±1.3 Hz), words (1.8±3 Hz), syllables (2.8±4.8 Hz), and phonemes (8±12.4 Hz); 2) and stronger that speech tracking responses for perceptually correct (comprehended) trials was only evident in the temporal lobe and motor cortex. Further, motoric cortical speech tracking was evident at the phrasal level corresponding to the delta beta band phase amplitude coupling indicating top-down temporal prediction in ongoing speech perception. In a study using connectivity measures for speech perception without concurrent sounds Park and colleagues [58] found that frontal top-down signals increased the coupling between auditory low-frequency oscillations and continuous speech: the entertainment of auditory cortical oscillations to speech (speech-brain coupling) increased with higher speech intelligibility and stronger low-frequency connectivity has been observed between the frontal and auditory cortices during intelligible than unintelligible speech, which also correlated with the strength of speech-brain coupling. These results are compatible with the notion that delta-theta brain oscillations play a role in implementing predictive control of the frontal cortex over the processing of continuous speech. An fMRI based FC study compared the effects of voluntary (top-down) and involuntary shifts of attention to sound events [59]. It was found that voluntary attention shifts were associated with stronger FC in the dorsal fronto-parietal network while stimulus (novelty) driven effects were accompanied by stronger FC in the ventral fronto-parietal pathway. Another fMRI study [60] reported that stronger FC between higher order cortical areas (such as the medial-lateral prefrontal cortices) facilitated comprehension of words with low semantic predictability when the intelligibility of speech was reduced. These results suggest that large-scale connectivity in higher-order brain areas may be involved in controlling auditory cortex during speech perception.

### Hypotheses

The stimulus paradigm of the current study allowed us to assess the whole brain functional networks involved in focusing one’s attention on a single speech stream in the presence of a second speech stream in contrast to following two concurrent speech streams. We fully crossed this manipulation with a task manipulation, which required integration of speech information along three different levels/time-scales: lexical detection (word level integration), syntactic violation detection (grammatical phrase– 2-3-word level integration), and content tracking (integration across multiple sentences). This unique experimental design allowed us to assess the brain networks underlying speech processing on different time-scales/depth and their interactions with the allocation of attentional resources. Please, note that the present study focused on the analysis on functional brain networks as opposed to speech-brain coupling.

Listeners were presented with two concurrent continuous speech streams while EEG and NIRS were simultaneously recorded. Because the present paper primarily focused on role of neural oscillations in different frequency ranges accompanying selective listening the NIRS data is reported only in supplementary material (S5, S6 and S8 Files, S6 Table, S2 and S3 Figs). Two types of tasks were employed in parallel: 1) the “tracking task", in which participants were instructed to follow the contents of one or both speech streams and 2) the “detection task” in which participants were to press a response key whenever they noted a numeral or a syntactic violation within the target speech stream. The target stream of the tracking task was either the same as that of the detection task (“focused attention condition”) or the opposite stream (“divided attention condition”). The attention and task type conditions were fully crossed. Based on the results of previous studies, we hypothesized higher FC strength in the dorsal fronto-parietal attention control network during the focused than the divided attention condition in the delta/theta [6,43], alpha [43], and possibly even in the beta band [48]. Unfortunately, little is known about functional networks during attention divided between speech streams. We assumed that processing information from two concurrent speech streams would result in higher demands on low-level speech processing steps and their attentional control. This would be evidenced by an increase of FC strength in a distributed network involving the posterior temporo-parietal cortex.

As described above, performing the three different tasks required integration of information from different units of speech having different time windows (from a few hundred milliseconds to several seconds). As these timescales correspond to different EEG bands, we hypothesized that the different tasks will activate networks operating in the bands corresponding to the length of the time windows within which information is integrated. That is, we expected to find stronger FCs for brain regions involved in speech comprehension in rather slow EEG bands (corresponding to information integration over long time windows) during the tracking task, while stronger FCs in the frequency bands (corresponding to integration of information within shorter time windows) in regions supporting lexical and syntactic processing during the detection tasks. (Ideally, one could even separate the two detection tasks this way, but in the current study, detecting syntactic violations required integration of maximum three short words (S1 File), which may not be separable from the single-word window in terms of EEG bands.)

## Materials and methods

### Participants

26 healthy young native Hungarian speaker adults (12 male, 14 female, mean age: 21.88 years, SD: 2.05; 24 right-handed) participated in the study for modest financial compensation. None of them had a history of psychiatric or neurological symptoms. All participants had pure-tone thresholds was ranging from 250 Hz to 4 kHz: <25 dB and <10 dB difference between ears. Informed consent was signed by all participants after the aims and methods of the study were explained to them. The study was conducted in full accordance with the World Medical Association Helsinki Declaration and all applicable national laws; it was approved by the institutional review board, the United Ethical Review Committee for Research in Psychology (EPKEB). One participant’s data were excluded from the EEG analysis due to >2 malfunctioning EEG channels.

### Stimuli

Participants listened to two concurrent streams of continuous Hungarian speech of ca. 6 minutes duration, each (mean duration: 352.15 s, SD: 9.34; mean word count: 636.41, SD: 84.87; mean number phonemes per word: 6.48, SD: 0.29) presented from two loudspeakers positioned symmetrically at 30° left and right from the frontal midline, 200 cm from the participant. The speech material was selected from a collection of news articles, which were reviewed by a dramaturge for correct grammar, natural text flow, and to prevent garden-path sentences. The information from which the articles were created was found on Hungarian news websites. The articles contained emotionally neutral, unfamiliar information. They were recorded from two male native Hungarian actors (20 articles, each) and edited by a professional radio technician. The soundtracks were recorded at 48 kHz with 32-bit resolution and presented by Matlab R2014a software (Mathworks Inc.) on an Intel Core i5 PC with ESI Julia 24-bit 192 kHz sound card connected to Mackie MR5 mk3 Powered Studio Monitor loudspeakers. The speech segments were recorded in the same room where the experiment took place and they were delivered from approximately the same location where the actor sat during the recording session (i.e., the loudspeaker was placed at the approximate location of the actor’s head). This confounded the location (side) of the loudspeaker and the identity of the speaker because all articles from the same actor were recorded at the same location. EEG recordings were synchronized to a beep sound preceding speech onset by 1 s. Each article contained 45–57 numerals (M = 50.7, SD = 2.7) consisting of 2–4 syllables. 32 of the 40 articles also included 19–26 (M = 20.5, SD = 1.4) syntactic violations. The distance between the mismatching words (subject-predicate or object-predicate) never exceeded 4 syllables (maximum two words). For a more detailed description of the syntactic violations, see S1 File).

The average timescales for phonemes syllables, words, phrases were quantified by first labeling them with WEBMAUS automatic segmentation tool [61] and hand-correcting in Praat [62]. Due to the limitations of WEBMAUS, only word, syllable, and phoneme segmentations were processed automatically; phrase segments were marked by expert inspection. Beyond the structure of the Hungarian language, average durations also reflect the speakers’ speech rate. The average duration of phrases for the first speaker was 3019 ms (standard deviation 1108 ms) and for the second speaker 3316 ms (std. 1440). The average duration of the words was 424 ms (std. 247 ms) for the first speaker 430,2 ms (std. 252 ms) and for the second speaker 418 ms (std. 241 ms). The average duration of syllables for the first speaker was 247 (std 160) and the second speaker 239 (std 154). The average length of phonemes was for the first speaker 106 ms (std. 37ms) and the second speaker 71 ms (std. 36ms).

### Procedure

Listeners were tested in an acoustically attenuated, electrically shielded, dimly lit room at the Research Centre for Natural Sciences, MTA, Budapest, Hungary. A 19” monitor was placed directly in front of the listener at a distance of 195 cm. Participants were instructed to keep eye blinks and all other motor activity to a minimum during the stimulus blocks by focusing on a fixation cross (the “+” sign) that was continuously present at the center of the monitor. For each stimulus block, two different articles (one for each speaker) were randomly selected for simultaneous presentation. Thus, participants were listening to two concurrent speech streams produced by two different speakers from two spatial locations.

**Fig 1:**
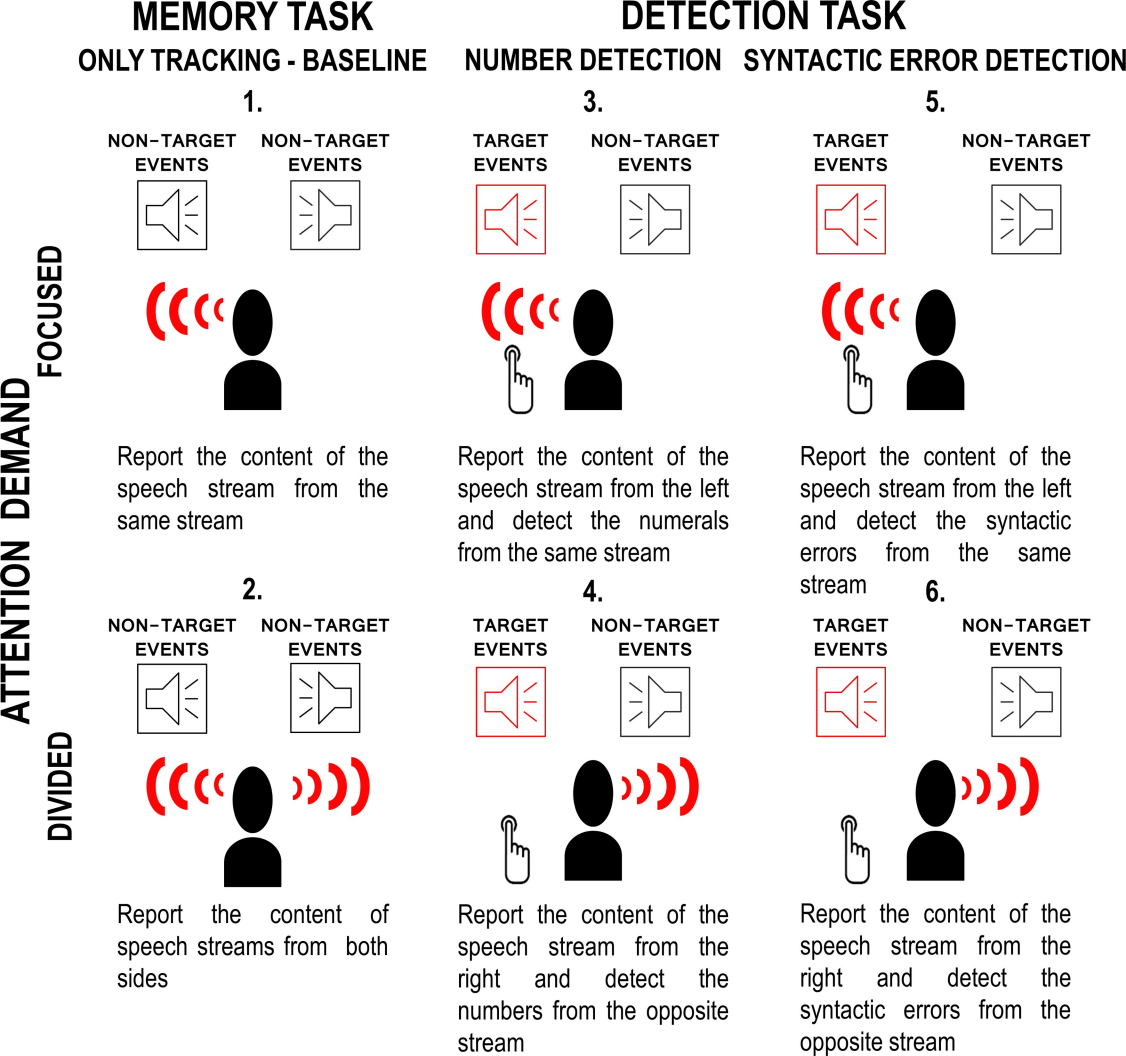
Schematic illustration of the six experimental conditions. Participants were listening to two concurrent speech streams under six experimental conditions: 1) Focused attention—only tracking (baseline) 2) Divided attention—only tracking (baseline) 3) Focused attention—numeral detection task 4) Divided attention—numeral detection task 5) Focused attention–syntactic violation detection task 6) Divided attention—syntactic violation detection task (see main text for the definition of the conditions). The gist of the task instructions specifying the target events for the detection task and the location of the target speech streams for each task are shown separately below each condition. The red “wave” pictograms indicate the target speech stream of the tracking task. Red “loudspeaker” pictograms and the “button-press” pictograms indicate the target speech stream of the detection task. Events like the targets of the detection task, when appearing in the non-target speech stream, served as distractors (non-target events).

Six experimental conditions were delivered (see Fig 1) in which combinations of three different tasks were employed. For the “tracking/memory task”, listeners were informed that at the end of the stimulus block, they will be asked 5 questions regarding the contents of one or both of the speech streams. When only one of the speech streams was task-relevant, it was identified by the side from which it was presented. The other two tasks (“detection task”) required real-time responses from the listener. They were asked to press a hand-held response key with their right thumb as soon as they detected the presence of a numeral target (“numeral detection task”) or a syntactic violation (“syntactic violation detection task”). Only numerals indicating the quantity of something within the context of the text were valid targets, words including a numeral as a part were not. The instruction for the syntactic violation detection task emphasized that the button should be pressed as soon as the listener detects that the sentence is grammatically incorrect. The assignment of the side of the detection tasks was constant within each listener, and it was counterbalanced across listeners. The tracking task was employed in each condition. There was either no other task (“only tracking”) or one of the detection tasks was employed. In the only tracking task conditions, the articles contained no syntactic violations. The target speech stream of the tracking task was either the same as that of the detection task (“focused attention condition”) or the opposite (“divided attention condition”). For the baseline of the divided attention condition, the speech streams from both sides were asked to be tracked (i.e., questions were asked about the contents of both streams). As a result, the following six task conditions were employed in the study (Fig 1): 1) Focused attention—only tracking task, 2) Divided attention—only tracking task, 3) Focused attention—numeral detection task, 4) Divided attention—numeral detection task, 5) Focused attention–syntactic violation detection task, 6) Divided attention—syntactic violation detection task. Note that in this arrangement, there is one target event (numeral or syntactic violation appearing in the stream designated for the detection task) and three types of non-target events. For disambiguation, we term the target events appearing in the concurrent stream as distractors. The other two non-target events are termed task-irrelevant events: syntactic violations appearing during the numeral detection task and numerals appearing during the syntactic violation detection task. Task-irrelevant events have been delivered both within the stream designated for the detection task as well as within the concurrent stream.

The two only tracking task conditions received 2 stimulus blocks, each, the other four conditions received 4 blocks, each. Thus, the experimental session consisted of 20 blocks, with one mandatory break after the 10th block and occasional shorter breaks between blocks as requested by the participant. The blocks for the focused attention only tracking task condition were presented at the 1st and the 20th position, those for the divided attention only tracking task condition at the 2nd and 19th position. The rest of the stimulus blocks were divided into two halves, each containing two blocks of each condition and they were delivered in a pseudorandomized order with the constraint eliminating consecutive stimulus blocks of the same condition. The articles with no syntactic violations were randomly assigned for each participant to one of the baseline conditions (separately for the two speakers) and similarly, the articles with syntactic violations were randomly assigned to one of the other task conditions (again, separately for each speaker).

After each stimulus block, a recognition memory test was performed (the test for the tracking task). The test consisted of 5 multiple-choice questions with 4 possible answers, each. Each question corresponded to one piece of information that appeared within the text assigned to the tracking task. The experimenter read the question and the 4 possible answers and the listeners were asked to verbally indicate the correct answer. The experimenter noted the participant’s choice and followed up with a request for confidence judgement with four possible options: “I don’t remember I was just guessing”, “I am not sure, but the option I chose sounded familiar; I think I heard it during the last block”, “I am sure; I remember hearing it during the last block”, “I know the answer from some other source”. The confidence judgment was then recorded by the experimenter.

### Data analysis

#### Behavioral responses

Detection task performance. Hits were initially searched for within a window of 0–5000 ms from the onset of the target events: onset of the numeral word or the onset of the word at which the syntactic violation could be detected. In order to exclude responses, which were unlikely to have corresponded to the given event, separately for the two detection tasks, responses were rejected if they were longer than 95% (>1885 ms for numerals and >2214 ms for syntactic violations) or shorter than 5% (<453 ms for numerals and <513 ms for syntactic violations) of all responses (collapsed across the divided attention conditions and participants). From the remaining responses, mean reaction times were calculated separately for each participant, detection task (numeral vs. syntactic violation), and attention condition (focused vs. divided). Next d‘ values (the standard measure for detection sensitivity; [63]) were calculated from the accepted responses („hits”) and the number of target events with no valid response („misses”). For „false alarms” and „correct rejections” time windows identical to the ones used for detecting hits were set for each distractor event (i.e., events of the same type occurring in the concurrent speech stream). The distractor effect was separately characterized by the ratio between the false alarms (i.e., responses to distractors) and all non-target responses (i.e., responses neither categorized as hits nor as false alarms), separately for each condition.

Tracking task performance. Recognition performance was separately calculated for each participant and condition. In order to increase the sensitivity of this measure, items (questions) with an overall correct response rate (collapsed across conditions and participants) above 95% or below 30% (25% representing chance level) were excluded from the analyses. No items needed to be dropped due to >95% correct response rate; 7 items with <30% response rate were discarded from the total of 160 items. Note that due to the random assignment of the texts across the different conditions, the same text (and thus the same questions) could have appeared in different attention/detection-task conditions for different participants. Further, responses with the confidence judgment “I know the answer from some other source” were excluded from the calculation of recognition performance measure for the given participant. Recognition performance was then calculated as the percentage of correct responses pooled across the stimulus blocks, separately for the four experimental conditions and participants.

#### EEG recording and preprocessing

EEG was continuously recorded during the presentation of the two concurrent speech streams with a BrainAmp DC 64-channel EEG system with actiCAP active electrodes. Electrodes were placed according to the International 10/20 system with the addition of one electrode placed on the tip of the nose. For EOG monitoring, one additional electrode was placed lateral to the outer canthus of the right eye and another below the left eye. Electrode impedances were kept below 15 kΩ. During the recording, the FCz lead served as the reference electrode. The sampling rate was 1 kHz, and a 100 Hz online low-pass filter was applied. Note that NIRS signals were recorded in parallel with the EEG. NIRS-related methods (recording and analysis) and results are presented in S5, S6 and S8 Files, S6 Table, S2 and S3 Figs.

**Fig 2:**
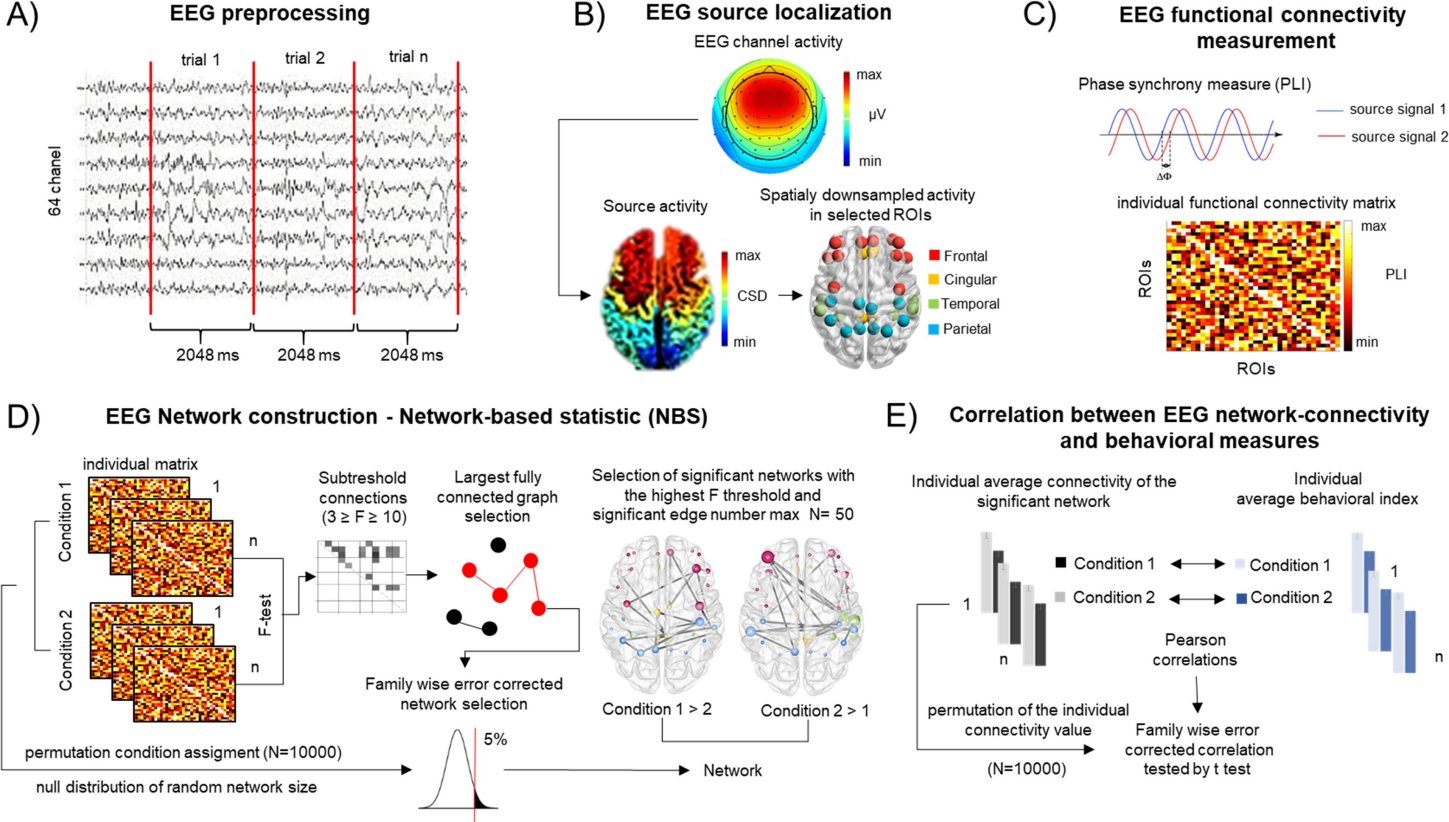
Schematic illustration of the data analysis. **A) EEG preprocessing**. Following primary filtering and ICA based artefact removal data was segmented. Epoch including target or non-target events or responses were excluded. Next, secondary artifact rejection (using threshold of 100 μV) was performed; **B) EEG source localization.** A minimum norm estimates model (sLORETA) for source-reconstruction was used together with forward boundary element head model (based on default anatomy and EEG locations); Current source density source activity was reconstructed for each voxel defined by the standardized parcellation scheme introduced by Klein & Tourville (2012). Finally, time-varying source signals was spatially down- sampled to the selected 36 ROIs; **C) EEG functional connectivity measurement.** Data were filtered for five frequency bands (from delta to gamma) and the phase lag index (PLI) was calculated as a measure of phase synchronization between each ROIs time series yielding 36 × 36 functional connectivity matrices for each individual, condition, and frequency band; **D) EEG Network construction—Network-based statistic (NBS).** An F test of each experimental contrast was run for each connection, and above-threshold connections selected for an F range between 3 and 10. The algorithm then searched for the largest fully connected network on each threshold level. Distribution of network size was pulled from the permutation of condition assignments (N = 10000). Family-wise error corrected p values of each network were obtained by comparing the network to the distribution derived from the random networks. Finally, the significant network on the highest F threshold level with only significant edges was selected (the maximum number of edges was set to 50); These networks were divided into two subnetworks according to the direction of the contrast effect. **E) Correlation between EEG network-connectivity and behavioral measures.** The average connectivity of the significant network emerging from each statistical contrast was correlated with the average behavioral indices (d, RT, recognition performance) of the corresponding condition. Family-wise error was controlled for each behavioral variable by estimating the distribution of the correlation coefficients via permuting the values of the network strengths 10,000 times.

An overview of the EEG data analysis pipeline is shown on Fig 2. EEG data analysis was performed using Matlab 2013a. The continuous EEG signal was off-line band-pass filtered between 0.5 and 45 Hz by a finite impulse response (FIR) filter (Kaiser windowed, Kaiser β = 5.65, filter length 4530 points) by the EEGlab 11.0.3.1.b toolbox (Delmore et. al., 2007). Maximum two bad EEG channels per participant were interpolated using the spline interpolation algorithm implemented in EEGlab. The Infomax algorithm of Independent Component Analysis (ICA) implemented in EEGlab was employed for artifact removal [64]. ICA components constituting blink artifacts were removed via visual inspection of their topographical distribution and frequency contents of the components.

Epochs of 2048 ms duration were extracted from the continuous EEG record as an epoch length of 2 sec affords optimal trade-off between the number of epochs with event and artifact-free trials (min 95 epochs per participant/condition). Based on previous studies, this epoch length is sufficient for assessing even low-frequency oscillatory activity [65,66]. Although longer epoch length generally results in lower connectivity values [66,67], this should not affect the contrasts tested in the study. Epochs including detection task targets or a button press were rejected from further analysis (note that the event-related brain potentials are reported separately: [68]. Epochs with an amplitude change exceeding 100 μV at any electrode were also rejected (Fig 2A). As a result, the dataset analyzed consisted of a minimum number of 95 epochs per participant/condition (mean: 161.8, SD = 26.2). The number of artifact-free epochs, separately for each condition, were: 132.24 in the baseline-focused attention task, 124.28 in the baseline-divided attention task (only two stimulus blocks, each), 174.6 in the focused attention-numeral detection task, 177.4 in the divided attention-numeral detection task, 181.88 in the focused attention-syntactic violation detection task, and 180.28 in the divided attention-syntactic violation detection task. Because stable and robust pattern of functional connectivity measure can be achieved from altogether 100 s of recording [69], therefore, regardless of the difference between the number of epochs contributing to the different conditions, the maximum number of epoch were included in the analysis for achieving the best possible signal to noise ratio.

#### EEG source localization

For source reconstruction (Fig 2B), a minimum norm estimate model (sLORETA developed by [70]) was applied by using the Brainstorm toolbox ([71]) with the protocol based on previous studies [72–77]. The MNI anatomical brain template was segmented using the default setting, as suggested by the Brainstorm tutorial [71]: 15002 voxels located in grey matter with a resolution of 1x1x1 mm. The default electrode locations were entered into the forward boundary element head model provided by the openMEEG algorithm [78]; the head model was based on a default anatomy derived from theMNI/Colin27 brain [79]. The time-varying source signals were modeled in all cortical voxels where the dipole had a component perpendicular to the cortical surface. By averaging dipole strengths across voxels, mean neuronal activity (current density) was obtained for the 62 cortical regions described by the standardized parcellation scheme introduced by [80]. 22 left- and 22 right-hemispheric cortical regions were selected for further analysis (occipital cortical areas were excluded from the further analysis; for list of the selected cortical regions with their abbreviations, see S1 Table). Because using the default anatomical template and electrode locations neglects inter-individual differences in head shape and electrode placement, source localization errors were evaluated for each cortical region of interest (ROI). The error assessment protocol and the results are reported in the S2 File (see, also S1 Fig and S2 Table). In summary, there are only few pairs of neighboring ROIs for which the reconstructed source activity could be ambiguous (5 pairs using 15 mm estimated error and 17 with using 20 mm estimate). The most affected regions are the Heschl or pars orbitalis, pars-triangularis and rostral and caudal anterior cingulate gyrus. We, therefore, do not draw conclusions basing on FCs including these regions.

#### EEG functional connectivity (FC) analysis

Phase synchronization strength as measured by the phase lag index (PLI; see [81] was calculated between each pair of EEG source region in five frequency bands (delta: 0.5–4 Hz; theta: 4–8 Hz, lower alpha: 8–10 Hz, upper alpha: 10–12 Hz, beta: 13–30 Hz, gamma 30–45 Hz following the band limits defined by [81]), separately for each EEG epoch (Fig 2C). It can be expressed in the following way:

$$PLI=|\langle sign[\Delta \varphi (t_{k})]\rangle |$$

where Δ*φ*(*t_k_*) refers to the time series of phase differences (t) calculated over all k = 1…N time points of a trial, *sign* refers to the signum function, ‹› refers to the mean value, and ││ denotes the absolute value. PLI is expressed as a value between 0 (random phase difference: minimum strength of FC) and 1 (constant phase difference: maximum strength of functional connectivity). In the current study, PLI (using default settings, gain = 1) was calculated by using the BrainWave software (version 0.9.151.5). Thus 44×44 weighted adjacency FC matrices were obtained for each epoch. FC matrices were then averaged separately for each participant, condition, and frequency band. Visualization of FCs on circular graph plots was performed by a Matlab function developed by Paul Kassebaum (available at http://www.mathworks.com/matlabcentral/fileexchange/48576-circulargraph). Visualization of the results of the FC analysis over the cortical surface was performed by the BrainNet Viewer toolbox [82]. For visualization of cortical surface, the BrainMesh_ICBM152 surface template was applied to the nodes representing the cortical gyri, which were located by their standard MNI coordinates.

### Statistical analysis

#### Behavioral data

Separately for d‘, RT, distractor effect, and the recognition index, repeated-measures ANOVAs were performed with the factors of DETECTION TASK (numeral vs. syntactic violation detection) × ATTENTION (focused vs. divided attention) × LOCATION (left vs. right detection task target stream), where Detection Task and Attention were within-subject, whereas Location between-subject factors. Statistical analysis was performed using the Statistics and Machine Learning Toolbox 10.1 of Matlab 2015b. The p-values of post-hoc pair-wise comparisons were adjusted using Bonferroni’s correction. All significant effects are described together with the η_p_^2^ effect sizes.

#### Functional connectivity data

The Network-based statistic (NBS) software package, developed for testing hypotheses about the human connectome [83] was used for statistical analysis. The NBS method exploits the tendency for experimental effects involving brain connectivity to exhibit specific topological characteristics that could not occur by chance in the absence of an effect (see Fig 2D;–for more details of the NBS analysis, see S3 File). The following effects of the experimental manipulations on FC strength were tested by pairwise NBS based statistical contrasts: **1)** the effect of the TASK TYPE: whether baseline (only tracking) and detection task (detection + tracking) FC networks are different—for this contrast, FC strength data were pooled for the two only tracking task and separately for all detection task conditions; **2)** the effect of ATTENTION: whether focused and divided attention condition FC networks are different—for this contrast, FC strength data were pooled for the focused and separately for the divided attention conditions. Two further contrasts were employed to test the effect of 1) the DETECTION TASK TYPE (whether the numeral and the syntactic detection task are different) and 2) the LOCATION (effect of location of the the task-relevant speech stream). The results of these analyses are reported in the S9 and S10 Files and S3 Table. The robustness of the observed network differences was assessed by calculating the effect size Cohen d’ separately for each identified network effects (S4 File).

#### Correlation between behavioral and EEG FC measures

In order to determine whether the processes indexed by FC are related to the inter-individual variance in task performance, correlations were calculated between the average connectivity strength values of the significant networks emerging from each statistical contrast and the average behavioral indices measured for each condition (see Fig 2E). FC strength for this analysis was calculated as the average of the strength values of the edges comprising the significant network selected through the NBS statistic. These average network strength values were then separetaly correlated with each corresponding behavioral variable (d’, RT, and recognition performance values) by Pearson’s correlation. The variables met all statistical prerequisites for performing permutation-based correlations. The family-wise error was controlled for each behavioral variable, separately, estimating the distribution of the correlation coefficients by permuting the network strengths values 10,000 times. From each permutation, the highest (absolute) correlation coefficient was extracted and the p-value was established as the proportion of these correlation coefficients that were higher than or equal to the observed coefficient. The list of correlations and their corresponding families are given in S3 Table. For the TASK-TYPE contrast-based correlation analyses, only recognition memory performance variables were correlated with the corresponding network strength variables, because recognition memory is the only behavioral variable that can be measured in both the only tracking and the detection task conditions (e.g., recognition memory performance after the divided attention detection task condition was correlated with the average strength of those divided attention condition networks, the edges of which were stronger for the divided attention detection task than for the tracking-only condition).

## Results

### Behavioral responses

**Fig 3:**
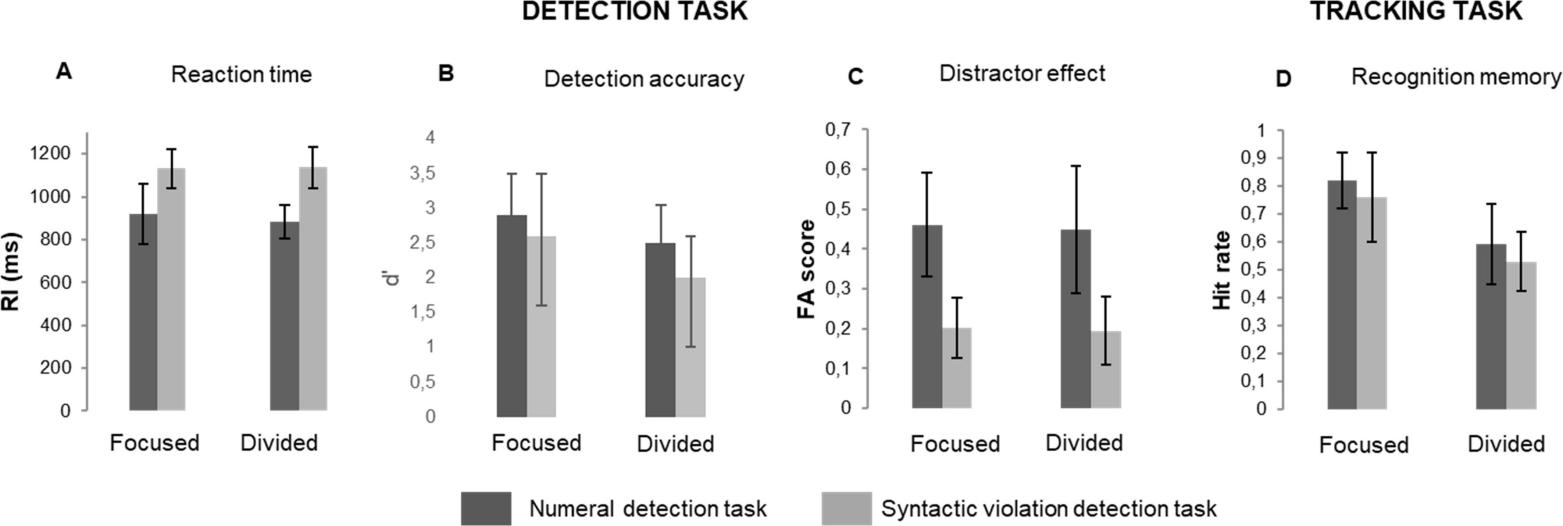
Group average (N = 25) performance in the detection (indexed by RT and d’; panels A and B, respectively), the ratio of detection task FAs (responses elicited by distractors) to the total of the non-target responses (i.e., responses neither categorized as hits nor as false alarms; panel C), and performance in the tracking task (recognition memory performance; panel D), separately for the numeral (dark grey bars) and the syntactic violation task (light grey bars) and for the focused (left) and divided attention condition (right). Line bars represent standard errors.

Fig 3 shows a summary of the behavioral measures. The behavioral results have also been described in the paper reporting the ERP effects [68]. For the effects of stream location on the performance measures, see S7 File.

#### Detection task performance

Analysis of d’ revealed significant main effects of DETECTION TASK (F1,24 = 9.235; p = 0.005, η_p_^2^ = 0.277) and ATTENTION (F1,24 = 18.951; p<0.001, η_p_^2^ = 0.441). Listeners performed significantly better with the numeral than the syntactic violation detection task and in the focused than the divided condition. The distractor effect was significantly larger with numeral than syntactic violation detection (main effect of the DETECTION TASK: F1,24 = 158.009; p<0.001, η_p_^2^ = 0.868). For RTs, the significant main effect of DETECTION TASK (F1,24 = 198.095; p<0.001, η_p_^2^ = 0.892) was due to listeners responding faster when detecting numerals than syntactic violations. However, because RTs were calculated from word onset, the RT difference between the two tasks may be biased by different delays from word onset for recognizing numerals compared to detecting a syntactic violation.

#### Tracking task performance

For the proportion of the correct answers to the questions asking about information related in the news articles, significant main effects of ATTENTION (F1,24 = 97.153; p<0.001 η_p_^2^ = 0.802) and DETECTION TASK (F1,24 = 11.258; p<0.005, η_p_^2^ = 0.319) were found. More details of the speech stream were remembered by listeners in the focused than the divided attention condition, and with the numeral than the syntactic violation detection task. The proportion of the correct responses in the tracking task only condition (not included in the above ANOVA) was higher for the focused attention (mean = 77.5; SD = 12.5) relative to the divided attention condition (mean = 64.9; SD = 14.2). No significant memory performance difference was found between the tracking task only and the detection task conditions.

### Functional networks

The statistical contrast yielded EEG functional networks significantly affected by ATTENTION (focused vs divided attended) as well as ones significantly affected by TASK TYPE (only tracking task vs. detection task) in two slow (delta: 0.5–4 Hz, and lower alpha 8–10 Hz), and one fast band of oscillations (beta: 13–30 Hz). These results will be detailed in the next two sections. According to the post-hoc analyses the effect of ATTENTION and TASK conditions on connectivity were ranging from medium to large (see S4 Table). S5 Table shows the summary of the EEG node degrees (the number of connections within networks showing a significant ATTENTION or TASK TYPE effect), separately for each ROI. Nodes with the highest degree could be identified as centers (termed as hubs) of interregional communication. The networks characteristically differ from each other by the hub locations and the relative contribution of the different lobes.

#### The effect of task type on functional connectivity

**Fig 4:**
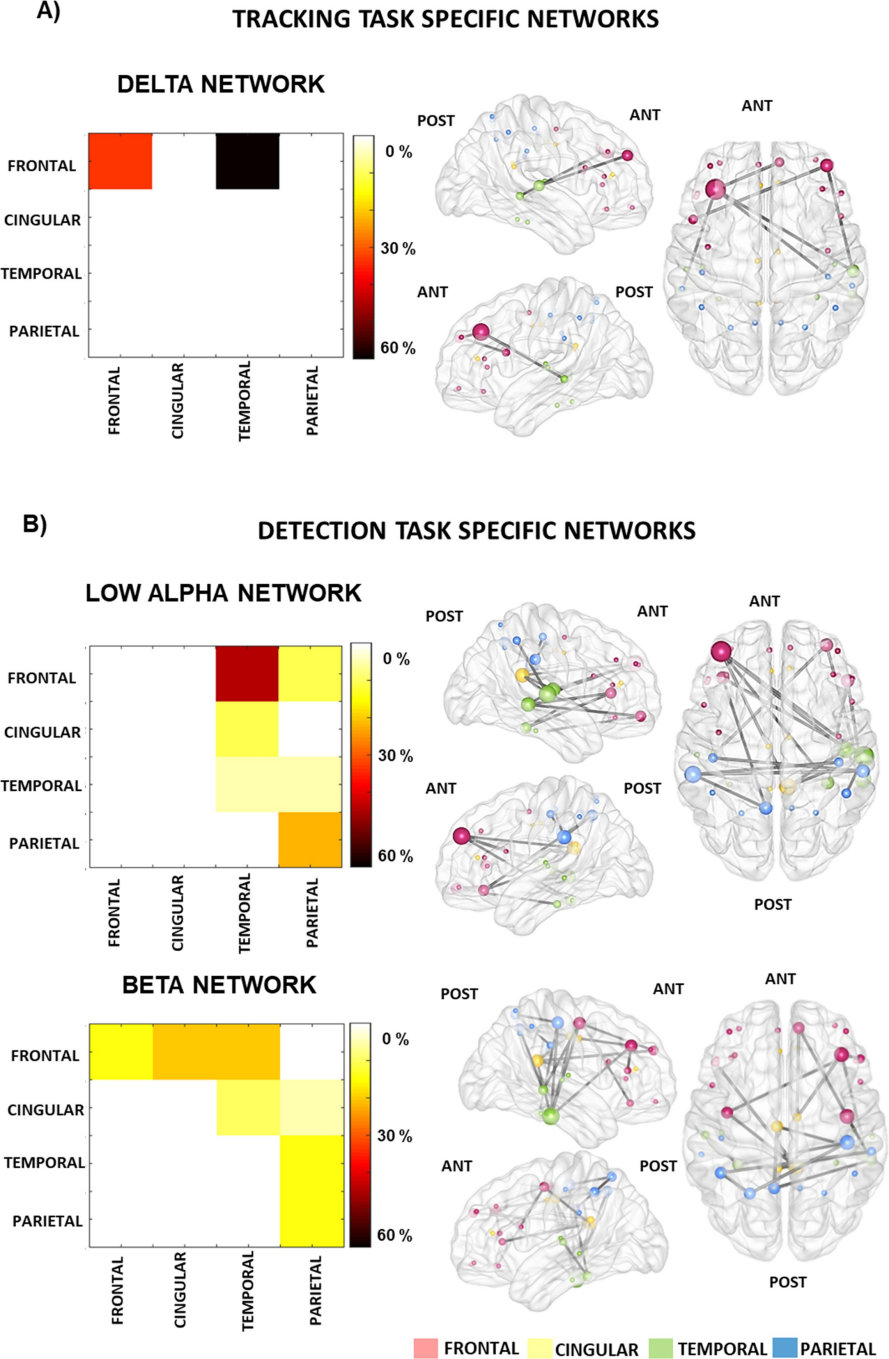
FC networks significantly affected by TASK TYPE: stronger for the tracking than for the detection task (Tracking Task Specific Networks: delta band panel A) and stronger for the detection than for the tracking task (Detection Task Specific Networks: EEG low alpha and beta bands panel B). The left column of panels A) and B) separately shows the regional distribution of the functional connections (color scale right from each panel). 100% refers to the sum of the connections comprising the significant network. The relative distributions of the connections are calculated for frontal, cingular, temporal and parietal cortices pooling the two hemispheres data. Values are plotted only above the diagonal. The right column of panels A) and B) separately shows a visualization of the significant networks on a plot of the cortical surface (top, left, and right view). Dots represent the spatial locations of the EEG sources reconstructed for cortical regions (nodes) in MNI space. The colors of the nodes indicate the cortical lobe: red–frontal; yellow–cingular; green–temporal; blue–parietal cortex. The size of the node represents the degree (number of connections within the network) of each node (see S5 Table).

Fig 4 shows the significant effects of the TASK TYPE manipulation for the delta, low alpha, and beta frequency bands. Regional distribution of the significant connections is depicted on the matrix panels, while a visualization of the significant networks is shown on a plot of the cortical surface.

*Delta band*.—Fig 4A, top. In the delta band all edges (N = 6; connecting 7 nodes) showed significantly (p<0.05) stronger connectivity during the tracking task relative to the detection task (TASK TYPE effect; K = 9.7, the threshold used in the F statistics–see S3 File; p = 0.048). The connections are predominantly within the frontal cortices and between frontal and temporal regions. Nodes with the highest number of connections (degree) within this network (S5 Table) were the MFG (caudal and rostral), and the medial temporal cortices (STG and MTG).

*Low alpha band*.***—***Fig 4B, top. All edges showed significantly (p<0.05, all) stronger connectivity during the detection task relative to the tracking task (TASK TYPE effect; K = 10.0; p = 0.004). The distribution predominantly involves the parietal cortices on both sides with some fronto-temporal connections. Nodes with the highest degree (S5 Table) were distributed across the frontal (MFG, OFG) temporal (HES, STG, MTG) and parietal (SMG, PoCG) cortices.

*Beta band*.***—***Fig 4B, bottom. All edges showed significantly (p<0.05, all) stronger connectivity during the detection task relative to the tracking task (TASK TYPE (K = 6.4; p = 0.047). The network can be characterized as comprising mainly fronto-temporal pathways mediated by the cingular cortices, as well as temporo-parietal and intra-parietal links. Nodes with the highest degree (S5 Table) were distributed across all four of the cortical areas assessed: cingular (ACC, PCG), frontal (PreCG, MFG), temporal (ITG, FFG), and parietal (IPG, PoCG).

#### The effect of focused vs. divided attention on EEG functional connectivity

**Fig 5:**
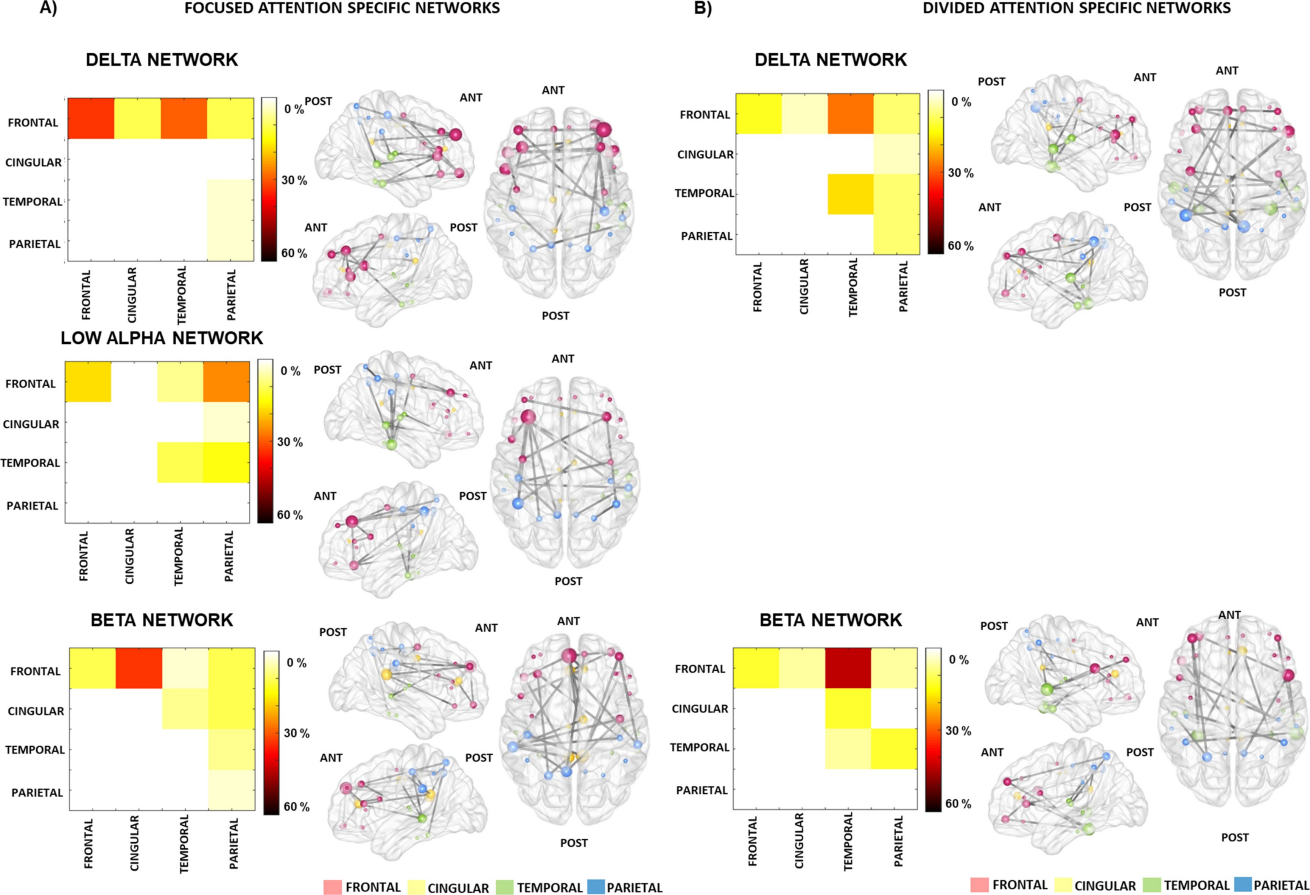
FC networks significantly affected by ATTENTION: stronger for focused than for divided attention (Focused Attention Specific Networks: EEG delta, low alpha, and beta bands; left: A) and stronger for divided than for focused attention (Divided Attention Specific Networks: EEG delta and beta bands; right: B). The left column of panels A) and B), separately shows the regional distribution of the functional connections (color scale right from each panel). 100% refers to the sum of the connections comprising the significant network. The relative distributions of the connections are calculated for frontal, cingular, temporal and parietal cortices pooling the two hemispheres’ data. For the sake of simplicity, the values are plotted only above the diagonal. The right column of panels A) and B), separately shows a visualization of the significant networks on a plot of the cortical surface (top, left, and right view). Dots represent the spatial locations of the EEG sources reconstructed for cortical regions (nodes) in MNI space. The colors of the nodes indicate the cortical lobe: red–frontal, yellow–cingular, green–temporal, blue–parietal cortex. The size of the node represents the degree (number of connections within the network) of each node (see S5 Table).

Fig 5 shows the significant effects of the ATTENTION manipulation, separately for the two possible directions of the effects (focused > divided attention and *vice versa*) in the delta, low alpha, and beta frequency bands. Regional distribution of the significant connections is depicted on the matrix panels, while a visualization of the significant networks is shown on a plot of the cortical surface.

*Delta band*.- Fig 5A, top shows those connections (N = 25 edges connecting 29 nodes) which were significantly (p<0.05, all) stronger during focused relative to divided attention (ATTENTION effect; K = 3.6; p = 0.047). Hub nodes were the IFG (N = 12) and the MFG (N = 11, see S5 Table). This network features functional connections within the frontal cortex (both within and across hemispheres) together with a few longer range fronto-temporal links.

Fig 5B top shows those connections (N = 22 connecting 23 nodes), which were significantly (p<0.05, all) stronger during divided relative to focused attention. These connections were primarily observed within temporal areas (both within and across hemispheres) and between frontal and parietal and frontal and temporal areas, mainly mediated through the frontal PreCG and IFG and the parietal IPG and PreCUN nodes (see S5 Table).

*Low alpha band-* Fig5A, middle shows those connections, which were significantly (p<0.05, all) stronger during focused relative to divided attention (ATTENTION effect: K = 4.3; p = 0.047; all but the connection between the right SFG and right HES). This network comprised 25 edges connecting 24 nodes. Nodes with the highest degree (S5 Table) were distributed across the frontal (MFG), temporal (ITG), and parietal (IPG) lobes. Characteristic connections included long-range fronto-parietal and local frontal connections.

*Beta band*.—Fig 5A, bottom shows those connections, which were significantly (p<0.05, all) stronger during focused relative to divided attention (ATTENTION effect: K = 0.1; p = 0.042). This network comprises 25 edges connecting 23 nodes. It mainly features functional connections (S5 Table) between the frontal and cingular cortices with relatively few local frontal and long-range fronto-parietal links (i.e. between SMG and MFG).

Fig 5B, bottom shows those connections, which were significantly (p<0.05, all) stronger during divided relative to focused attention. This network comprises 25 edges connecting 18 nodes and it mainly consists of fronto-temporal (frontal nodes of IFG and MFG with temporal nodes, such as MTG and FFG) and a few temporo-parietal connections (e.g., HES-IPG).

#### Correlation between behavioral and EEG FC measures

TASK-TYPE contrast based analyses: The average memory performance score in the divided attention only tracking task condition was significantly positively correlated with the average connection strength of the tracking task specific network in the delta band (r(25) = .530, pfwe = .025). The average memory performance score in the divided attention detection task condition was significantly positively correlated with the detection task specific network connectivity strength in the lower alpha band (r(25) = .496, pfwe = .043).

ATTENTION contrast based analyses: The average memory performance score of the divided attention conditions was significantly positively correlated with the strength of the beta band focused attention specific network (r(25) = .505, pfwe = .041).

## Discussion

Functional connectivity estimates were utilized for extracting whole brain functional networks operating on different timescales while listeners attended to one or two continuous simultaneously delivered speech streams. Brain networks in delta, low alpha, and beta frequency bands were differentially activated when listeners either focused on a single speech stream versus when they divided their attention between the two streams. The connectivity strength of brain networks also varied as a function of the duration of the time window within which information was integrated for successfully performing the task. Moreover, the strength of FC within these networks was associated with the task-performance measures. Thus, these networks may operate in parallel performing specialized functions. In the following, we separately address the observed effects on functional brain networks.

### Dynamic functional networks sensitive to the requirements of the listening task

**Fig 6:**
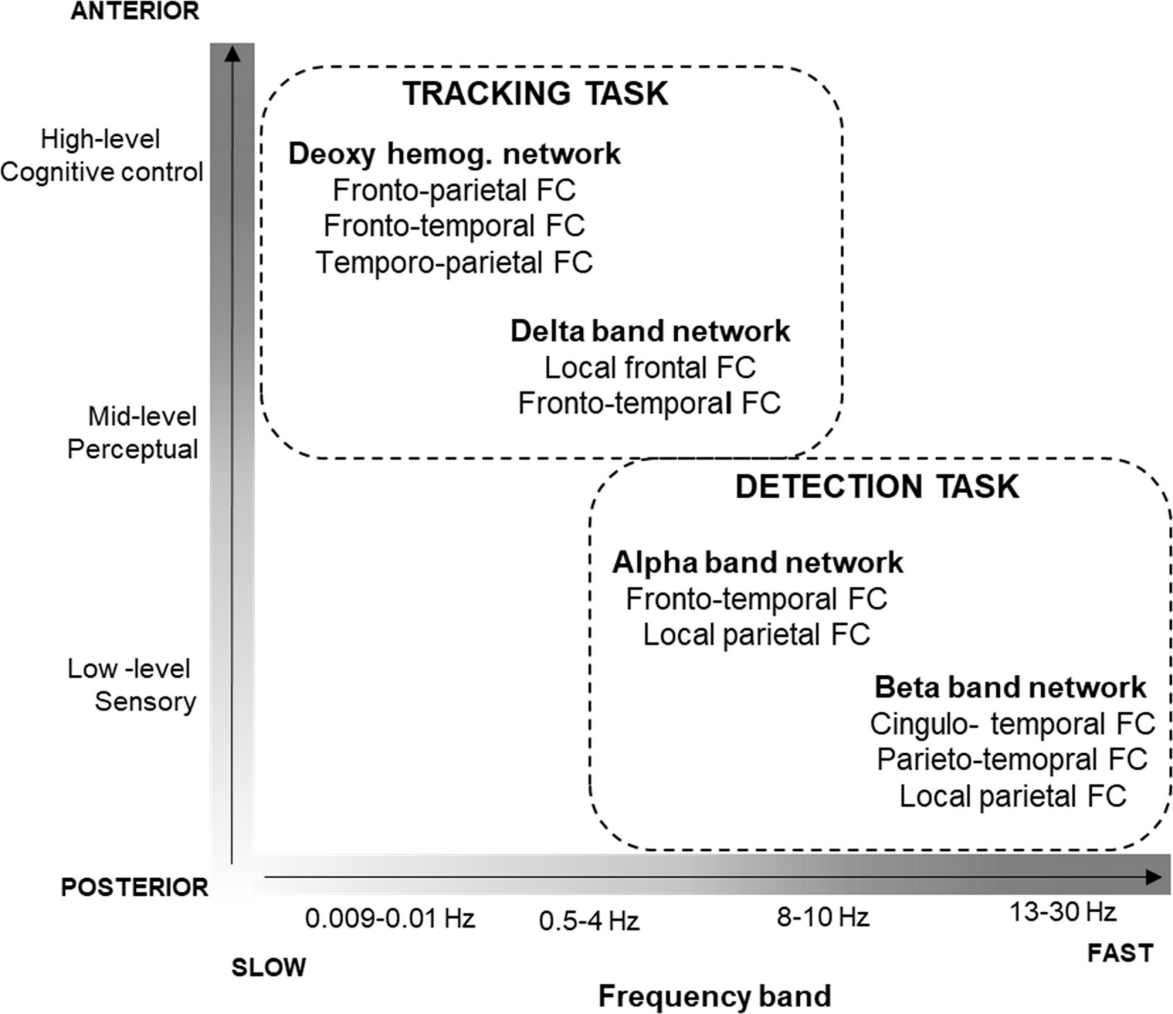
A schematic depiction of the potential roles of functional networks in sensitive to the TASK TYPE contrast as a function of the NIRS/EEG frequency bands and a schematic brain functional hierarchy. (Recording, analysis, and results for the NIRS data can be found in S5, S6 and S8 Files, S6 Table, S2 and S3 Figs). The NIRS/ EEG frequency scale is represented on the *x*-axis, whereas the functional hierarchy on the *y*-axis. The networks with stronger connectivity during the detection task are predominantly located in sensory/perceptual areas and operate on higher oscillatory frequencies, whereas the networks with stronger connectivity during the tracking task are located in perceptual/cognitive-control areas and operate on lower oscillatory frequencies.

We found that the tracking task induced stronger coupling within brain regions involved in speech comprehension in the slow delta frequency band of EEG (corresponding to information integration over long time windows of ca. 500–2000 ms duration) while the detection tasks were associated with stronger connectivity between regions supporting lexical and syntactic processing in the relatively faster alpha and beta bands (corresponding to integration of information within shorter time windows of ca. 33–125 ms duration). We also found that the tracking task relative to the detection task-induced stronger coupling of the slow hemodynamic and the EEG delta frequency bands in anterior brain regions (for a description of the recording, processing, and results of the NIRS data, see (S5, S6 and S8 Files, S6 Table, S2 and S3 Figs).). Our tracking task facilitated listeners to integrate longer periods of the speech segments (phrases lasting for ca. 3 seconds and longer) while encoding their information in longer-term memory. In contrast, the detection task promoted listeners to focus their attention on a shorter time scale, because the target events could be detected without reference to the longer context (i.e., by lexical analysis or analysis of grammatical structures involving 2–3 successive words, the numeral and the syntactic violation detection task, respectively) and an immediate response was required to them. The average word duration was ca. 425 ms; thus, 2–3 words were 850–1275 ms long. Thus, the relationship between phrase durations and the length of the delta cycles and that between 1–3 words and the alpha cycles is similar: 2–4 cycles of these oscillations would cover the period of the corresponding speech segments. We also note that with decreasing oscillation frequencies, the focal points of these networks appear to progress from areas responsible for sensory processing towards areas regarded as supporting high-level cognitive processes, such as contextual processing and cognitive control. Therefore, we tentatively suggest that the different oscillation frequency ranges may reflect differences in the length of the speech segments, whose information is integrated (e.g., in working memory) for performing the task. No FC difference was observed between numeral and syntactic-violation detection (see S9 File). This may have been due to the fact that detecting syntactic violations required integration of maximum three short words, which is not be separable from the single-word window in terms of the traditional EEG bands (a limitation of the current methods). Further, the lack of differentiation between the two types of detection tasks may also indicate that extracting and manipulating certain lexical and grammatical speech features may rely on largely overlapping neural networks. However, event-related phasic neural activity usually distinguishes these two aspects of speech [68,84,85] (. The networks showing significant effects of task type in our study are represented along their timescales and neuro-anatomical characteristics on Fig 6. A more detailed discussion of the networks sensitive to task type are given below.

#### Neural network supporting speech comprehension over longer time-scales

*The cerebral blood flow based network* (reported in S8 File): The network showing higher FC during tracking as opposed to target detection is left-lateralized and fronto-parietal with superior and lateral parietal and superior-medial and inferior frontal hubs. These nodes were more strongly coupled with each other and with medial temporal regions (medial and Heschl). The observed network largely overlaps with those parietal and frontal areas, which have been shown to support the integration of narrated stories over longer time scales [86,87]. In these neuroimaging studies, the hierarchy of the temporal windows of language processing was mapped in subjects listening to real life stories scrambled at the timescale of words, sentences, or paragraphs. It was found that longer timescales involved a succession of brain regions from low-level auditory to higher order language areas. Frontal regions appeared to accumulate information over the longest time windows (from the sentence level to the entire story; for a review see [88]). We therefore interpret the task-type sensitive network based on slow (0.009–0.01Hz) deoxyhemoglobin fluctuations as reflecting the activation of higher-order areas with long processing timescales (many seconds to minutes, which approximate the time course of a coherent segment within the news piece-like text; Fig 6.), as these cortical circuits can accumulate information at the highest level of the hierarchy affected within the current study.

*Delta band network (0*.*5–4 Hz)*: The core of this network comprised links within the frontal and between frontal and temporal cortices. Stronger involvement of higher-level frontal areas in the tracking task relative to detection task supports the notion that integrating information from longer windows during tracking task may rely on higher-order areas. Given the temporal spread of the delta waves, delta-band FC between frontal and temporal areas may support the integration of words into sentences (at the time scale of ca. 2 s; Fig 6). This functional role of the task-type sensitive low-frequency networks is also supported by the observed linear correlation between the strength of delta band FC and memory performance in the tracking task. Specifically, stronger FC was linked with better memory performance in the only tracking task condition. This suggests that these networks may reflect, at least in part, sustained memory-related processes (semantic information retrieval, episodic encoding, etc.) and sustained allocation of attention to the target speech stream. This idea is in line with the observation that central nodes of the networks which show stronger coupling for the tracking task alone are located in the frontal cortices in the MTG and parietal association cortices (SPG and SMG). The MTG has been shown to participate in accessing lexical, semantic, and conceptual information and the inferior and midline frontal cortices display extensive projections to the aforementioned temporal and parietal nodes implicated in contextual integration [11,89].

#### Neural network supporting linguistic analysis over shorter timescales

In contrast to tracking task, the detection task induced stronger connectivity in the alpha and beta frequency bands showing more distributed networks that included the sensory-perceptual brain regions: the detection task specific networks consist of connections within the temporal cortex and between temporal and parietal areas. Further, the low alpha band network strength correlated with memory performance. However, unlike for the low-frequency networks, here connectivity strength was related to the memory index only when participants performed a detection task (in addition to the tracking task). This indicates that faster oscillations may contribute to detection-task related processes and enhance memory performance through reducing the interference between the detection and the tracking task.

It is, however important to note that the functional role of alpha and beta oscillations in listening conditions are still controversial. Our interpretation that the frequency of the oscillation is related to the duration of the task-relevant speech segments is compatible with the suggestions that 1) beta-band activity is linked with top-down lexical semantic predictions such as lexical-semantic prediction of an upcoming word based on prior context [15] and 2) alpha oscillations are associated with the storage of syntactic phrases in verbal working memory for the downstream establishment of dependencies with other phrases [15]. These functions are central to the requirements of the detection task, whereas they are part of a larger set of functions required in the tracking task. Further, these functions require integration of information over shorter timescales compared to what is needed for successfully performing the tracking task.

An alternative to this interpretation can be based on the assumption that the detection task may require stronger auditory attentional control relative to the content tracking task, and alpha and beta band networks represent neural inhibition of the ignored auditory stream for better detecting targets in attended stream. In favor of this alternative, it was shown that 1) alpha activity may reflect the listener attending versus ignoring some speech input, which could be interpreted as protecting the processing of the attended speech signal from interference by suppressing the unattended input [42,43,90,91]; 2) beta band activity is assumed to be a good proxy of control processes in adverse listening conditions and auditory perceptual decision [44,46,92].

### The functional networks underlying focusing or dividing auditory attention

Listeners remembered more information from the speech segments in the focused than in the divided attention conditions. These results are consistent with previous reports on dichotic listening [51,93]. One possible way of interpreting the attention effect is that focusing attention on a single speaker allows deeper processing of the speech stream, while effectively suppressing the concurrent stream reduces the amount of information to be processed. In contrast, dividing attention across multiple speech streams results in shallower encoding and more information to be processed. The beneficial effects of focused attention could be due to improving the signal-to-noise ratio for task-relevant information either by enhancing gain on cortical activity related to the target and/or by suppressing the activity elicited by the task-irrelevant speech stream [4,5,7,8,94]. In contrast, dividing attention between two parallel speech streams may require sharing processing resources across them. This requires that a larger amount of data is processed in parallel, the maintenance/representation of two concurrent stream representations together with more intensive (finer-grained) top-down control over these processes.

**Fig 7:**
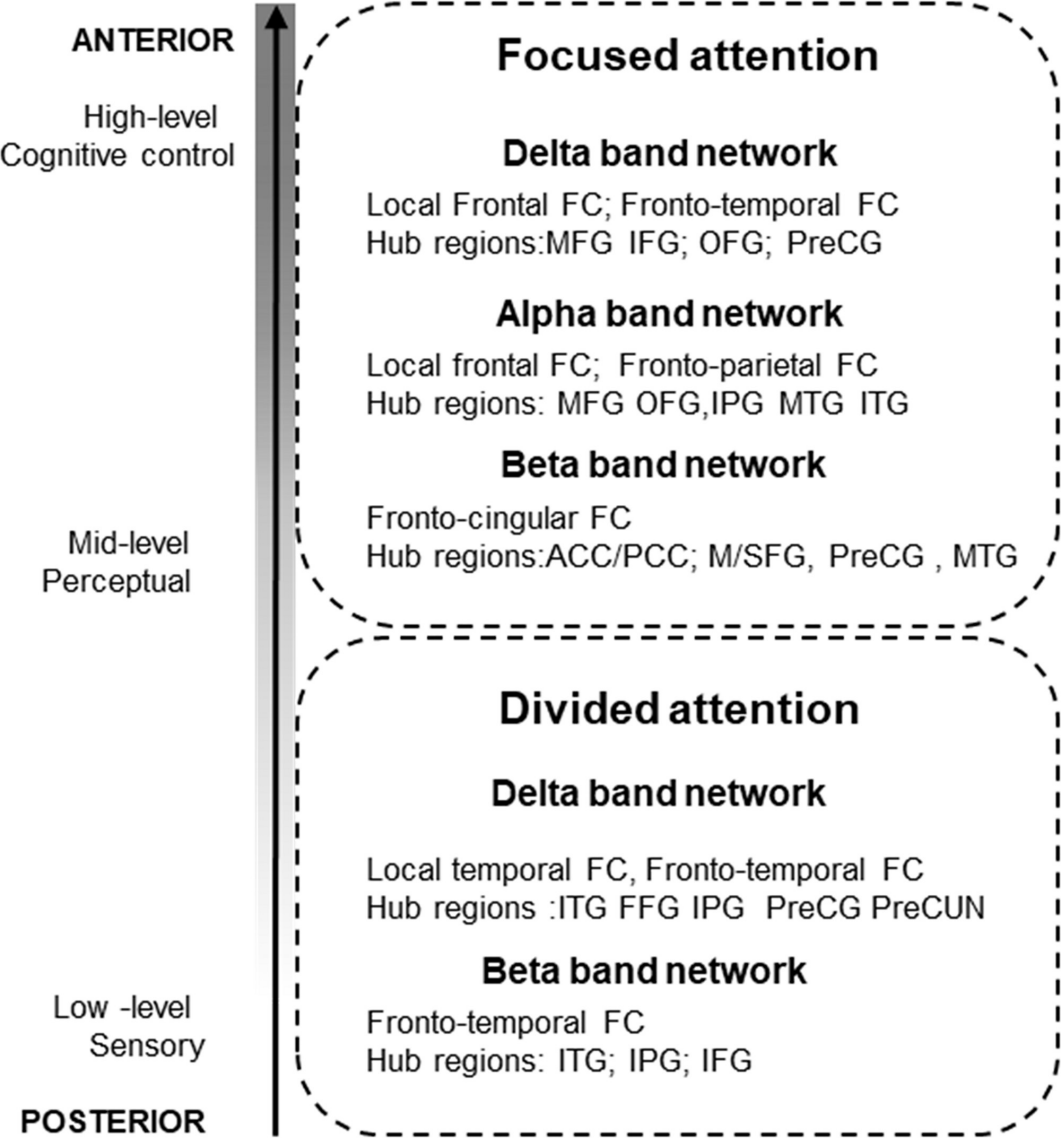
A schematic depiction of the potential roles of functional networks in mediating focused vs. divided attention during processing two concurrent speech streams. The y axis represents the functional level as well as the main hub regions of the networks.

In the present study we identified two separate sets of functional networks operating in parallel in multiple frequency bands, one associated with focused, the other with divided attention in our multi-talker situation. Because, for this analysis, we contrasted the two attention conditions, the networks emerging point out those parts of the attention system, which are specific to one or the other attentional situation. While the two sets of networks overlap in oscillation frequencies as well as in the main areas involved, the role of higher-order areas is prominent in the focused attention specific networks, whereas in the networks associated with divided attention, sensory/perceptual areas play a more important role. A summary of these networks is shown at Fig 7. Overall, connections strengthened by focused attention may reflect control over information selection, whereas connections strengthened by divided attention may reflect the need for maintaining two streams in parallel increased amount of speech processing and the related control processes necessary for performing the tasks. A more detailed description of the observed networks follows.

#### Functional networks specific to focused auditory attention

Many of the frontal areas involved in the networks showing stringer activation with focused in comparison with divided attention have been previously implicated in attentional control [9,95,96] and working memory control functions ([97] 003). Thus these networks may be involved in voluntary control of speech processing and information selection mediated by the dorsal fronto-parietal attentional system in the low (delta, theta; e.g., [94]), alpha (e.g., [43]), and in the beta band (e.g., [48]).

*Delta band network (0*.*5–4 Hz)*: This network is characterized by local frontal and fronto-temporal connections. We suggest that this network may play a role in enhancing the processing of task-relevant information because 1) regardless of the stimulus material, fronto-parietal phase synchronization in the delta band has been shown during sustained attention and this synchronization decreased with cognitive fatigue but increased during the orientation of attention [37,98] and 2) specifically, during speech perception, delta oscillations in auditory cortex are modulated by functional connections from higher-order areas (frontal and motor) to primary auditory cortex, thereby changing/controlling the auditory cortical entrainment to the speech input [35,38,43,58]. Therefore, the stronger coupling observed in the focused, as compared with divided attention in the delta band may reflect mechanisms enhancing the responsiveness/entrainment to the task-relevant speech stream.

*Alpha band network (8–10 Hz)*: We interpret our finding of stronger alpha-band FC between the midline frontal and parietal cortices during focused compared to divided attention as the mechanism of suppressing the task-irrelevant speech stream under the control of the MFG. Alpha-band activity has been previously associated with the suppression of task-irrelevant information during working memory and selective attention tasks ([60,99–102] with particular relevance in spatial auditory selective attention [42,43] and speech processing in the presence of maskers [42,56,103]. Current alpha-band network also exhibited stronger FCs within temporal cortices and projections from temporal to prefrontal and parietal cortices. Similar results were obtained in a MEG study [56], which showed that during dichotic listening to speech, functional coupling of the alpha rhythm increased between left auditory and Wernicke’s areas (Wernicke area is a part of SMG). This network calls into mind the well-known topography of the dorsal (STG projects dorsal-posterior toward inferior parietal and frontal lobe regions, such as premotor areas in PreCG) and the ventral (STG projections to MTL and the IFG, from IFG to PreCG) language pathways [11,89]. Thus, the current alpha-band network may also be related to the observation of posterior alpha activity while listeners maintained information about lateralized sounds in working memory [104] as well as to the higher inter-areal alpha-band phase synchronization found during demanding information processing that involved working memory [41]. Taken together, these findings suggest that selective attention may have enhanced both the ventral and the dorsal pathways for speech encoding through increased alpha-band posterior connections.

*Beta band network (13–30 Hz)*: The beta-band FC that were found to be stronger for focused than divided attention included links from the cingular cortices (i.e. ACC) to the frontal (PreCG, IFG, SFG, OFG) and posterior areas (SMG, PoCG, MTG). Also, we demonstrated that stronger connectivity of this network was accompanied by better subsequent memory accuracy. We interpret the observed beta-band network as possibly contributing to the prefrontal monitoring functions facilitating the local processing of task-relevant stimuli in posterior sensory association areas [46,105]. Specifically, this function may operate via supporting processing speech information through auditory sensory and motor areas. Further, the anterior part of cingular cortices (ACC) is also presumed to serve as a relay station between frontal speech producing regions (IFG) and it plays a critical role in the control of attention, and memory processes [106–108]. The involvement of the premotor region in the beta-band FC networks is compatible with the view that articulatory movements mediated by beta–band oscillations provide sensory-motor representation by internal motor simulation of the perceived phonemes [104,109–113].

#### Functional networks specific to divided auditory attention

*Delta band network (0*.*5–4 Hz)*: This network has connections within the temporal cortex and from the temporal cortex to the frontal and parietal cortices. This network largely overlaps with the fronto-temporo-parietal network observed for speech perception, a network that is known to be affected by the stimulus-driven allocation of auditory attention [10,59,96,114,115]. This may reflect that the increased the sensory information processing load of memory storage and stream monitoring is accompanied by an increase of FC strength in a distributed network involving the sensory/perceptual auditory areas while requiring more nuanced control from frontal areas with information possibly flowing both ways (top-down as well as bottom-up aspects of attention).

*Beta band network (13–30 Hz)*: This network connects sensory/perceptual areas of language processing with fronto-temporal and temporo-parietal links. These results are consistent with the reported involvement of IFG pars orbitalis (BA47) in semantic processing [11,89]. We suggest that the higher processing load caused by processing multiple speech streams during the divided attention conditions may be reflected by stronger coupling in beta-band functional networks with hub regions that are located in lower level sensory and perceptual areas (i.e. motor; inferior temporal; inferior parietal cortices) of the speech processing networks [110].

### Limitations

Although extracting phase synchronization (especially PLI), and the rigorous statistical techniques used to test experimental contrasts are aimed to eliminate the spurious connections resulting from volume conduction [116], EEG source localization still holds uncertainty because distributed source models such as sLORETA provides spatially smoothed solution based on probability estimation of the source distribution. Thus, neighborhood sources are conditioned to assume similar current density field strengths (the Laplacian regularization priors).

An additional limitation of the current study is the lack of individual’s structural anatomy by MRI or digitized EEG sensor locations [75]. According to reviews [74,77] LORETA is able to recover smoothly distributed sources with relatively low localization error. The error has been estimated by dual fMRI-EEG to approx. 14.5-16mm of the MRI activation focus. Simulating an EEG inverse source reconstruction for testing the consequence of using approximate head model [73] yielded a median localization error of 10.3 mm. One study indicated that localization errors of a spatio-temporal dipole fit in the head volume with the sphere model are on the range of 6 to 20mm with high rates of residual variance in the data [72].

Finally, it is important to note that the length of the analyzed epoch may influence the connectivity value. Here 2 sec long EEG segments were extracted that allowed us to maintain an optimally high number of epochs that are free of artefacts and transient evets. In other words, we aimed to keep the trade-off between the number of the epochs and the length of epochs optimal. It has been reported that longer epoch lengths generally result in lower connectivity values [66,67]. Although the different epoch lengths for EEG and NIRS may have influenced the magnitude of connectivity, the effects of conditions observed on EEG and NIRS networks would have to be independent of the connectivity magnitude.

### Summary

We found that 1) tracking the contents of long speech segments, which require information to be integrated for several seconds (induced by speech tracking task) was allocated stronger connectivity in lower frequencies (delta band), whereas linguistic analysis of speech in shorter timescales (induced by detection task) was linked with the network in the faster alpha and beta bands. Furthermore, the strength of network connectivity was found to be predictive for the task-performance measures. Therefore, these networks may operate in parallel performing specialized functions.

In the current study, we found that tracking the contents of long speech segments, which requires information to be integrated over several seconds, was associated with increased connectivity strength of EEG delta-band and even slower NIRS-signal based functional networks connecting areas involved in speech comprehension. In contrast, detection numerals and syntactic violations with maximum 4 syllables separating the grammatically linked words were associated with stronger connectivity in networks operating in the relatively faster EEG alpha and beta bands, connecting regions supporting lexical and syntactic processing. Furthermore, connectivity strength of these networks correlated with task-performance measures. We suggest that the frequency of these oscillatory networks may be related to the duration of the time windows from which information is integrated. We also found EEG delta-, alpha-, and beta-band functional networks with predominantly frontal hubs, which were associated with focused as compared to divided attention and delta- and beta-band networks with hubs in auditory sensory and perceptual areas linked with divided as compared to focused attention. These networks may reflect the differences in task demands between focusing or dividing one’s attention in a multi-talker situation (e.g., information selection vs. speech processing and maintaining two streams in parallel).

## Supporting information

S1 Fig Results of the assessment of source localization accuracy. ROI pair distances above the thresholds of 15 (top panel) and 20 mm (bottom panel) are plotted as lines connecting the corresponding ROI centers. The threshold was defined as 50% indicating that more than half of the ROI’s voxel’s source activity could be unreliably attributed to another ROI. The present EEG source localization solution could result in a high degree of overlap for 5 pairs (15 mm estimated error) or 17 pairs (20 mm estimated error) of ROIs.
(TIF)

S2 Fig NIRS channel and EEG electrode montage. NIRS sources (red dots), detectors (blue dots), and channels (green lines) with channel numbers printed over the line are shown for the configuration used in the experiment. Blue circles represent standard EEG electrode positions. Some of the NIRS optodes were slightly moved for reaching the optimal 3 cm distance between each source-detector pair (not marked on this Fig). NIRS channels were spatially clustered into the 11 left and 11 right-hemispheric cortical regions. The abbreviations of the NIRS cortical regions (see S1 Table) are indicated within the shaded regions of the plot. The color of shaded regions represents the large-scale brain areas with blue marking the parietal red the frontal, green the temporal region ROIs selected for the analyses.
(TIF)

S3 Fig NIRS FC networks significantly affected by TASK TYPE. Stronger for the tracking than for the detection task (Tracking Task Specific Networks. The left column of panels A) and B) separately shows the regional distribution of the functional connections (color scale right from each panel). 100% refers to the sum of the connections comprising the significant network. The relative distributions of the connections are calculated for frontal, cingular, temporal and parietal cortices pooling the two hemispheres data. Values are plotted only above the diagonal. The right column of panels A) and B) separately shows a visualization of the significant networks on a plot of the cortical surface (top, left, and right view). Dots represent the spatial locations of the EEG sources reconstructed for cortical regions (nodes) in MNI space. The colors of the nodes indicate the cortical lobe: red–frontal; yellow–cingular; green–temporal; blue–parietal cortex. The size of the node represents the degree (number of connections within the network) of each node (see S6 Table).
(TIF)

S4 Fig Grand average spectral power. Spectral density is shown for all 64 channel channels separately (colored lines). The scalp distribution of the power for 1 Hz, 10 Hz and 20 Hz are plotted above the diagram.
(TIF)

S1 Table Source regions and their abbreviation. Source regions and their abbreviation (third column) for EEG (second column) and NIRS sources (fourth column) grouped according to large-scale anatomical areas (first column).
(DOCX)

S2 Table EEG source localization accuracy results. All ROIs pairs (listed region as A-B) above the threshold degree of overlap reported separately for 15 and 20 mm localization error distance values.
(DOCX)

S3 Table Supplementary information on EEG FC and behavioral data correlation analysis. (DOCX)

S4 Table Results of the post hoc pairwise dependent sample t-tests performed on the average subnetwork connectivity strength values (Student’s t, degree of freedom, p, and Cohen’s d effect size values). (DOCX)

S5 Table Summary of the number of connections within the subnetworks that showed significant ATTENTION or TASK TYPE effects, separately for the three EEG bands (columns). Each line represents a ROI (identified by its abbreviation as defined in S1 Table). ROIs are grouped by brain lobes (Frontal, Cingular, Temporal, and Parietal). The sum of connections within each lobe and the percentage of connections relative to all connections within subnetworks are also listed.
(DOCX)

S6 Table Summary of the number of connections within the subnetworks showing a significant TASK TYPE effect for the NIRS deoxygenated hemoglobin concentration. Each line represents a ROI (identified by its abbreviation as defined in S1 Table). ROIs are grouped by brain lobes (Frontal, Cingular, Temporal, and Parietal). The sum of connections within each lobe and the percentage of connections relative to all connections within the subnetworks are also listed.
(DOCX)

S1 File Stimuli: Syntactic violation. (DOCX)

S2 File Assessment of EEG source localization accuracy. (DOCX)

S3 File Statistical analysis: Extended description of the NBS statistics. (DOCX)

S4 File Effect size measure of FC statistics. (DOCX)

S5 File NIRS recording and preprocessing. (DOCX)

S6 File NIRS functional connectivity analysis. (DOCX)

S7 File Supplementary results: Behavioral responses–testing the effects of location. (DOCX)

S8 File Supplementary results: NIRS deoxygenated hemoglobin concentration. (DOCX)

S9 File Supplementary results: The effect of the DETECTION TASK TYPE on EEG FC. (DOCX)

S10 File Supplementary results: The effect of the LOCATION. (DOCX)
