# Supplementary figures and images for: Attention and speech-processing related functional brain networks activated in a multi-speaker environment
Source: PLoS One. 2019 Feb 28;14(2):e0212754. doi: 10.1371/journal.pone.0212754 (PMC6394951; doi:10.1371/journal.pone.0212754)

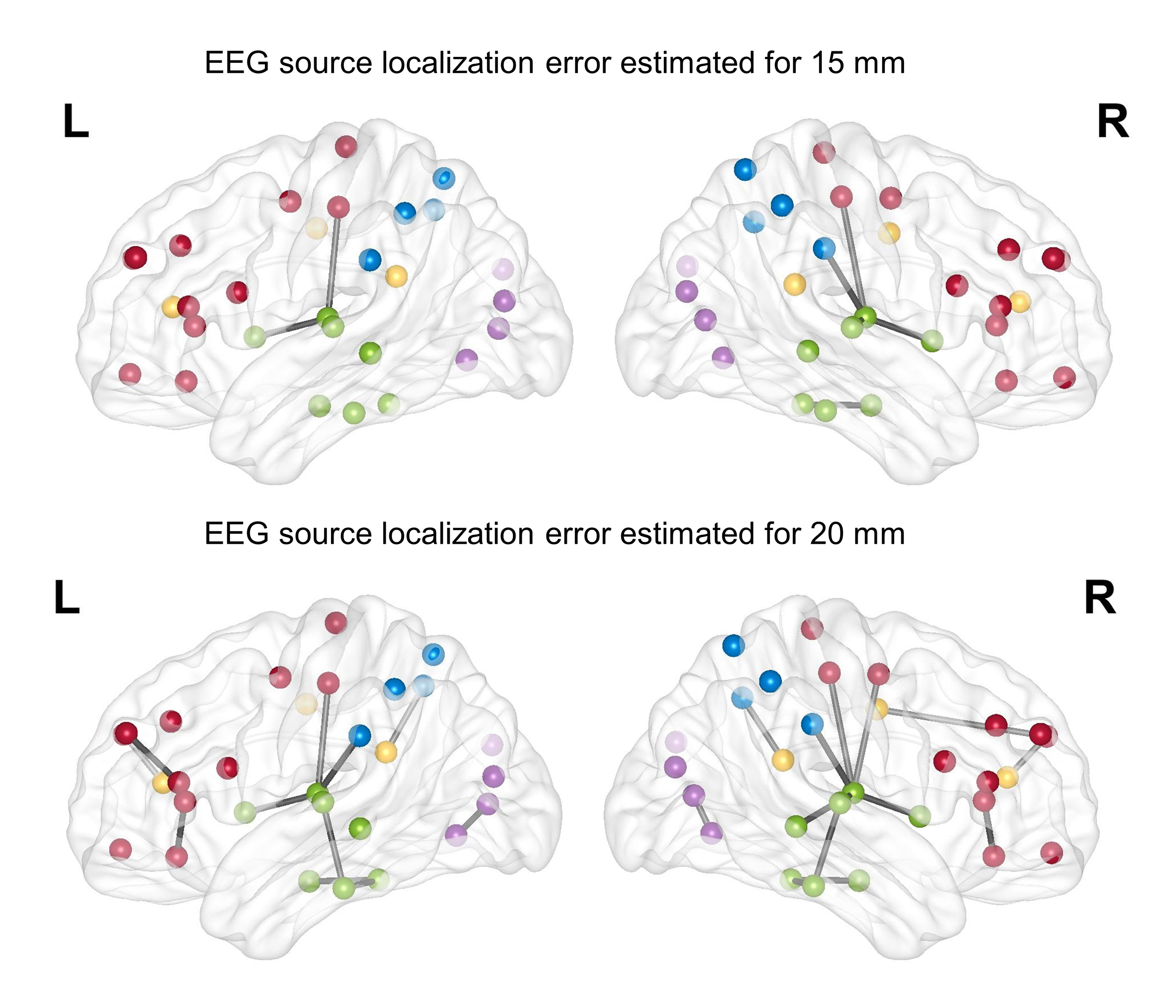

Supplement: S1 Fig — ROI pair distances above the thresholds of 15 (top panel) and 20 mm (bottom panel) are plotted as lines connecting the corresponding ROI centers. The threshold was defined as 50% indicating that more than half of the ROI’s voxel’s source activity could be unreliably attributed to another ROI. The present EEG source localization solution could result in a high degree of overlap for 5 pairs (15 mm estimated error) or 17 pairs (20 mm estimated error) of ROIs. (TIF) [file pone.0212754.s001.tif]

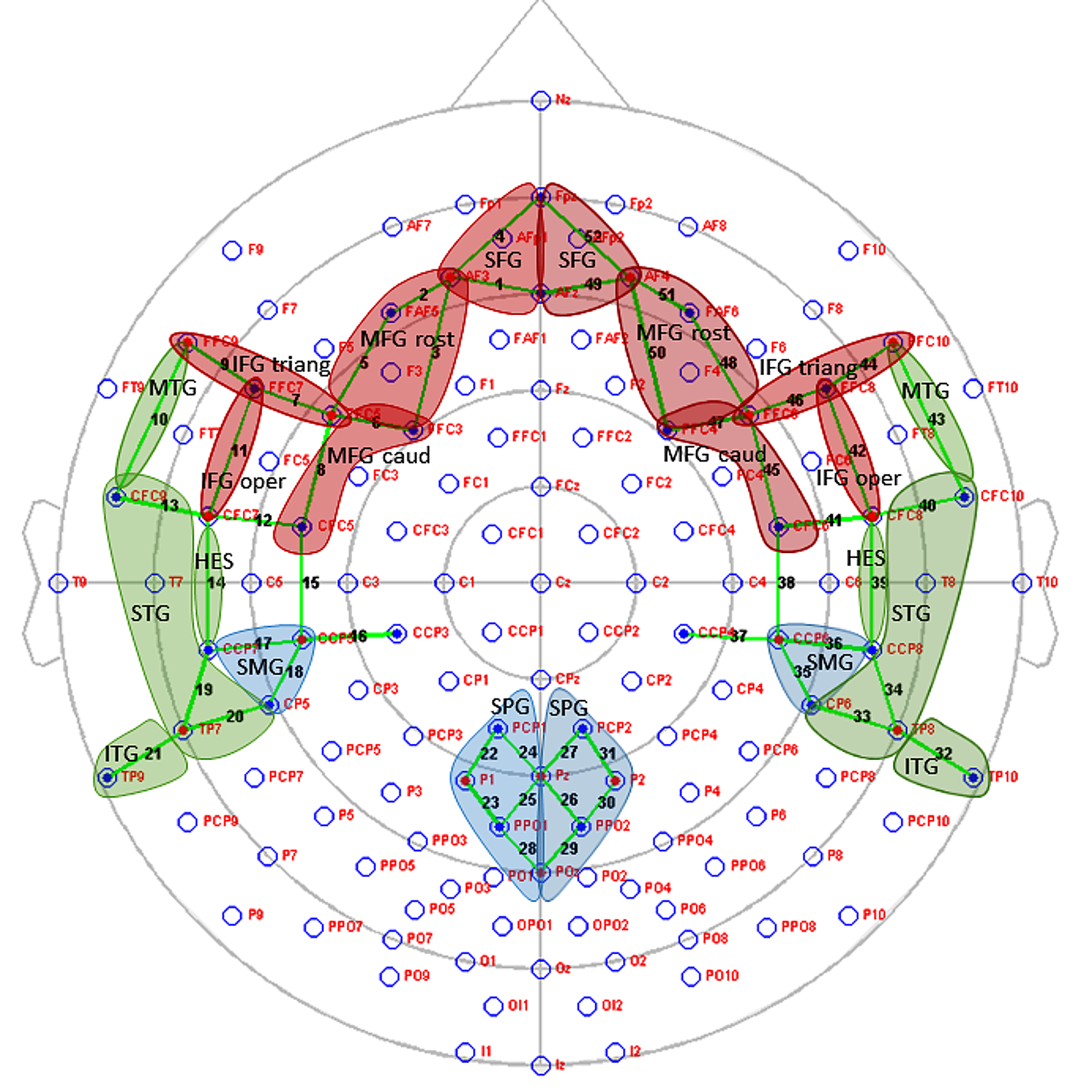

Supplement: S2 Fig — NIRS sources (red dots), detectors (blue dots), and channels (green lines) with channel numbers printed over the line are shown for the configuration used in the experiment. Blue circles represent standard EEG electrode positions. Some of the NIRS optodes were slightly moved for reaching the optimal 3 cm distance between each source-detector pair (not marked on this Fig). NIRS channels were spatially clustered into the 11 left and 11 right-hemispheric cortical regions. The abbreviations of the NIRS cortical regions (see S1 Table) are indicated within the shaded regions of the plot. The color of shaded regions represents the large-scale brain areas with blue marking the parietal red the frontal, green the temporal region ROIs selected for the analyses. (TIF) [file pone.0212754.s002.tif]

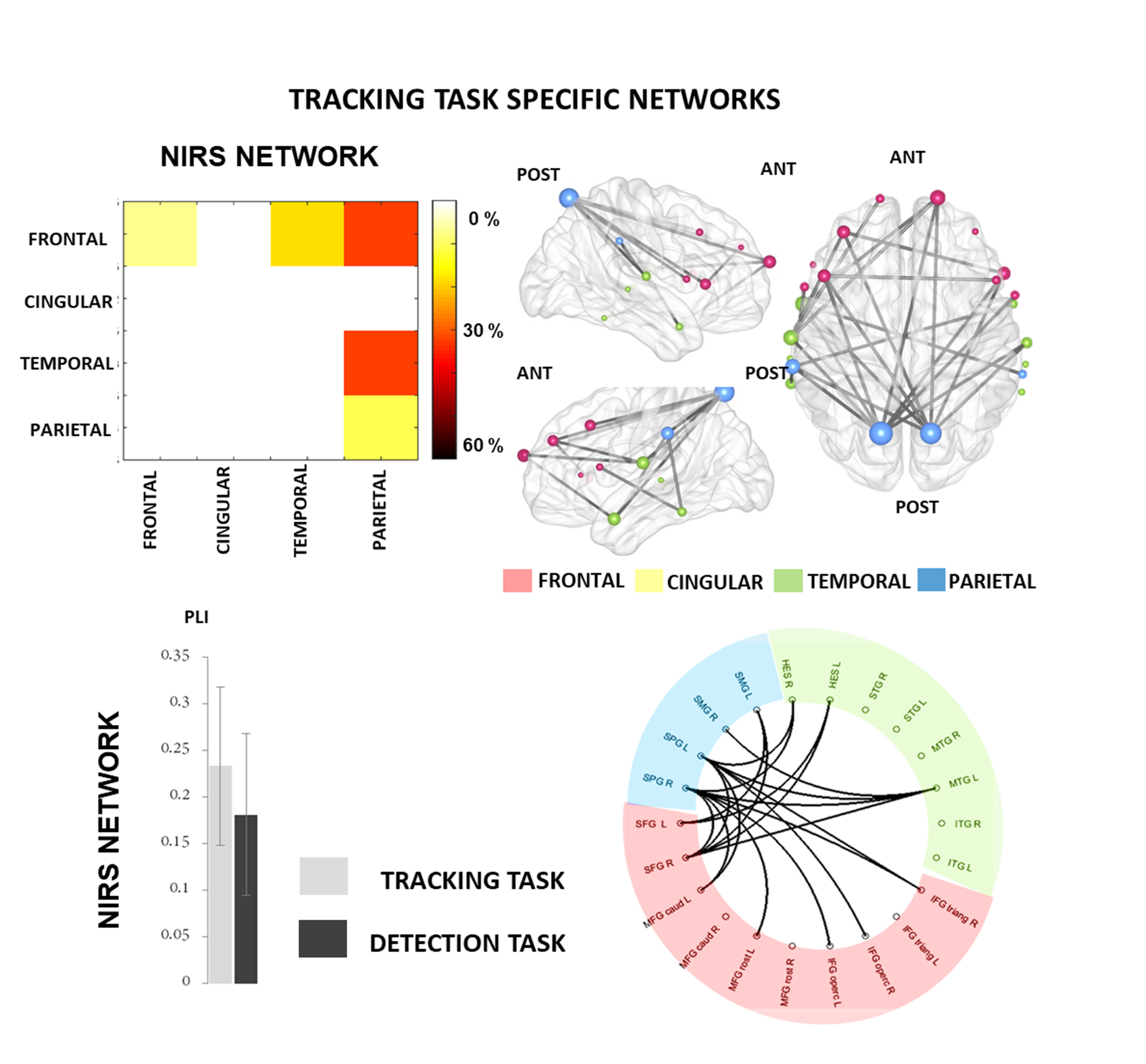

Supplement: S3 Fig — Stronger for the tracking than for the detection task (Tracking Task Specific Networks. The left column of panels A) and B) separately shows the regional distribution of the functional connections (color scale right from each panel). 100% refers to the sum of the connections comprising the significant network. The relative distributions of the connections are calculated for frontal, cingular, temporal and parietal cortices pooling the two hemispheres data. Values are plotted only above the diagonal. The right column of panels A) and B) separately shows a visualization of the significant networks on a plot of the cortical surface (top, left, and right view). Dots represent the spatial locations of the EEG sources reconstructed for cortical regions (nodes) in MNI space. The colors of the nodes indicate the cortical lobe: red–frontal; yellow–cingular; green–temporal; blue–parietal cortex. The size of the node represents the degree (number of connections within the network) of each node (see S6 Table). (TIF) [file pone.0212754.s003.tif]

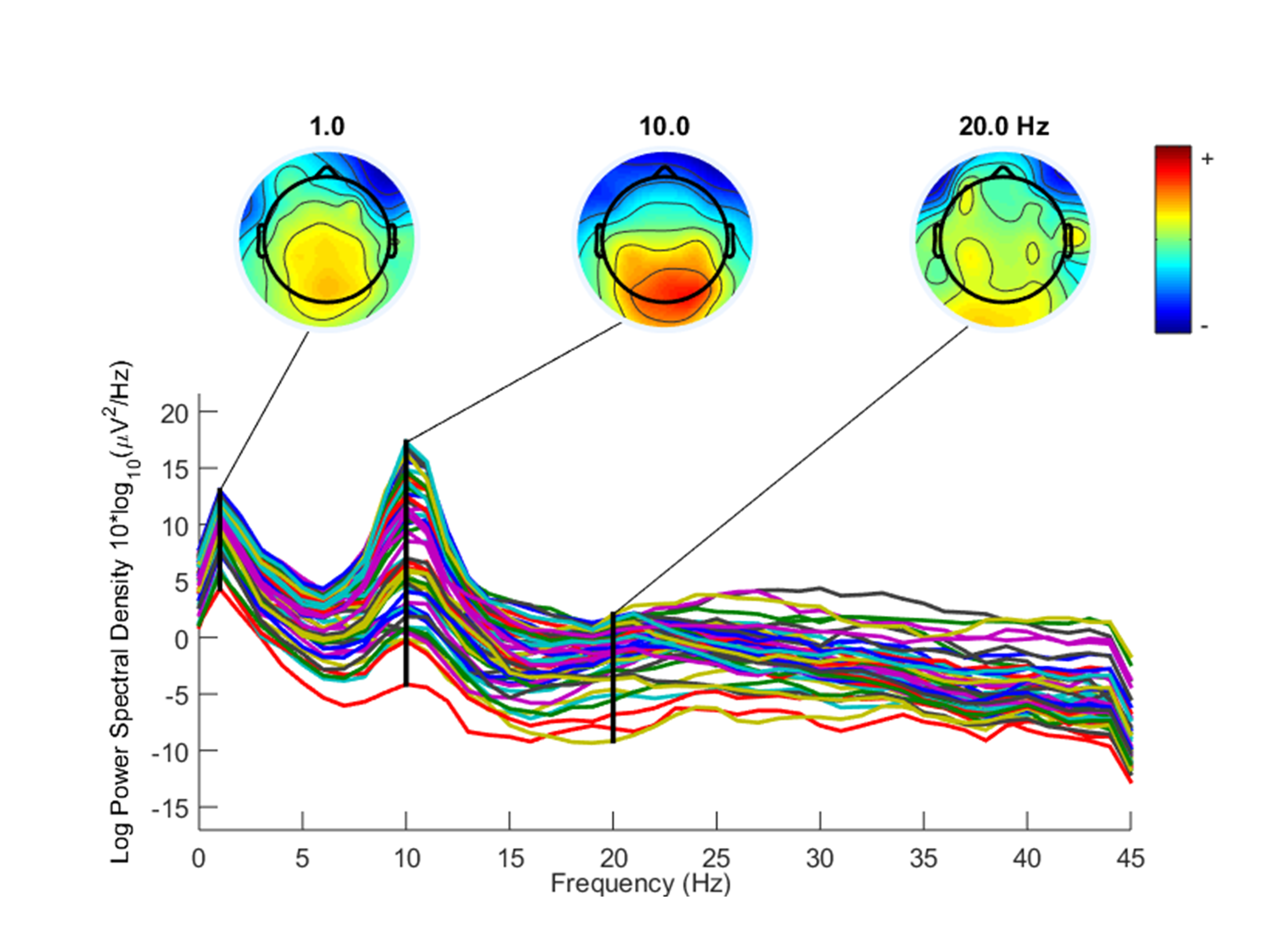

Supplement: S4 Fig — Spectral density is shown for all 64 channel channels separately (colored lines). The scalp distribution of the power for 1 Hz, 10 Hz and 20 Hz are plotted above the diagram. (TIF) [file pone.0212754.s004.tif]
