# Supplementary material for: Attention and speech-processing related functional brain networks activated in a multi-speaker environment
Source: PLoS One. 2019 Feb 28;14(2):e0212754. doi: 10.1371/journal.pone.0212754 (PMC6394951; doi:10.1371/journal.pone.0212754)
Supplement: S1 Table — Source regions and their abbreviation (third column) for EEG (second column) and NIRS sources (fourth column) grouped according to large-scale anatomical areas (first column). (DOCX) [file pone.0212754.s005.docx]

| Anatomical region | EEG source regions | Abbreviation | NIRS source regions |
| --- | --- | --- | --- |
| Frontal | Caudal Middle Frontal Gyrus | MFG caud | Caudal Middle Frontal Gyrus |
|  | Rostral Middle Frontal Gyrus | MFG rost | Rostral Middle Frontal Gyrus |
|  | Lateral OrbitoFrontal Gyrus | OFG lat |  |
|  | Medial OrbitoFrontal Gyrus | OFG med |  |
|  | Inferior Frontal Gyrus parsOpercularis | IFG operc | Inferior Frontal Gyrus parsOpercularis |
|  | Inferior Frontal Gyrus parsOrbitalis | IFG orb |  |
|  | Inferior Frontal Gyrus parsTriangularis | IFG triang | Inferior Frontal Gyrus parsTriangularis |
|  | Superior Frontal Gyrus | SFG | Superior Frontal Gyrus |
|  | PreCentral Gyrus | PreCG |  |
| Cingular | Caudal Anterior Cingulate Gyrus | ACG caud |  |
|  | Rostral Anterior Cingulate Gyrus | ACG rost |  |
|  | Posterior Cingulate Gyrus | PCG |  |
| Temporal | Fusiform Gyrus | FFG |  |
|  | Inferior Temporal Gyrus | ITG | Inferior Temporal Gyrus |
|  | Middle Temporal Gyrus | MTG | Middle Temporal Gyrus |
|  | Superior Temporal Gyrus | STG | Superior Temporal Gyrus |
|  | Heschl Gyrus | HES | Heschl Gyrus |
| Parietal | Inferior Parietal Gyrus | IPG |  |
|  | Superior Parietal Gyrus | SPG | Superior Parietal Gyrus |
|  | SupraMarginal Gyrus | SMG | SupraMarginal Gyrus |
|  | Postcentral gyrus | PoCG |  |
|  | Precuenus | PreCUN |  |
