# Supplementary material for: Attention and speech-processing related functional brain networks activated in a multi-speaker environment
Source: PLoS One. 2019 Feb 28;14(2):e0212754. doi: 10.1371/journal.pone.0212754 (PMC6394951; doi:10.1371/journal.pone.0212754)
Supplement: S2 Table — All ROIs pairs (listed region as A-B) above the threshold degree of overlap reported separately for 15 and 20 mm localization error distance values. (DOCX) [file pone.0212754.s006.docx]

| Region A | Region B | 1.5 cm | 2.0 cm |
| --- | --- | --- | --- |
| Heschl | supramarginal | 73.62 | 128.8 |
| Heschl | superiortemporal | 115.5 | 281.17 |
| Heschl | middletemporal |  | 51.82 |
| Heschl | precentral |  | 57.04 |
| Heschl | postcentral | 52.53 | 104.05 |
| Heschl | insula | 109.51 | 191.58 |
|  |  |  |  |
| parahippocampal | fusiform | 53.03 | 101.37 |
| entorhinal | fusiform |  | 85.21 |
| entorhinal | inferiortemporal |  | 102.43 |
| entorhinal | superiortemporal |  | 58.11 |
|  |  |  |  |
| rostralanteriorcingulate | superiorfrontal |  | 75.56 |
| rostralanteriorcingulate | rostralanteriorcingulate |  | 72.28 |
| caudalanteriorcingulate | superiorfrontal |  | 95.09 |
| caudalanteriorcingulate | caudalanteriorcingulate |  | 82.02 |
|  |  |  |  |
| parstriangularis | rostralmiddlefrontal |  | 50.11 |
| parsorbitalis | parstriangularis |  | 79.68 |
| parsorbitalis | lateralorbitofrontal |  | 80.77 |
|  |  |  |  |
