# Supplementary material for: Attention and speech-processing related functional brain networks activated in a multi-speaker environment
Source: PLoS One. 2019 Feb 28;14(2):e0212754. doi: 10.1371/journal.pone.0212754 (PMC6394951; doi:10.1371/journal.pone.0212754)
Supplement: S3 Table — (DOCX) [file pone.0212754.s007.docx]

| Individual average connectivity of the significant network | Individual average behavioral index | | |
| --- | --- | --- | --- |
| ATTENTION contrast-based correlation analyses | | | |
|  | **Family 1.** | **Family 2.** | **Family 3.** |
| Delta band focused attention | d' | RT | Recognition index |
|  | focused attention numeral detection task | focused attention numeral detection task | Focused attention numeral detection task |
|  | Focused attention syntactic violation detection task | focused attention syntactic violation detection task | Focused attention syntactic violation detection task |
| Delta band divided attention | d' | RT | Recognition index |
|  | in focused attention numeral detection task | in focused attention numeral detection task | in focused attention numeral detection task |
|  | in focused attention syntactic violation detection task | in focused attention syntactic violation detection task | in focused attention syntactic violation detection task |
| Low alpha band focused attention | d' | RT | Recognition index |
|  | in focused attention numeral detection task | in focused attention numeral detection task | in focused attention numeral detection task |
|  | in focused attention syntactic violation detection task | in focused attention syntactic violation detection task | in focused attention syntactic violation detection task |
| Beta band focused attention | d' | RT | Recognition index |
|  | in focused attention numeral detection task | in focused attention numeral detection task | in focused attention numeral detection task |
|  | in focused attention syntactic violation detection task | in focused attention syntactic violation detection task | in focused attention syntactic violation detection task |
| Beta band divided attention | d' | RT | Recognition index |
|  | in focused attention numeral detection task | in focused attention numeral detection task | in focused attention numeral detection task |
|  | in focused attention syntactic violation detection task | in focused attention syntactic violation detection task | in focused attention syntactic violation detection task |
| TASK-TYPE contrast-based correlation analyses | | | |
| Delta band tracking task | Recognition index |  |  |
|  | in focused attention only-tracking task |  |  |
|  | in divided attention only-tracking task |  |  |
|  | in focused attention detection task |  |  |
|  | in divided attention detection task |  |  |
| NIRS deoxyhemoglobin tracking task | Recognition index |  |  |
|  | in focused attention only-tracking task | | |
|  | in divided attention only-tracking task | |  |
|  | in focused attention detection task | | |
|  | in divided attention detection task | |  |
| Low alpha band detection task | Recognition index |  |  |
|  | in focused attention only-tracking task | |  |
|  | in divided attention only-tracking task | |  |
|  | in focused attention detection task | |  |
|  | in divided attention detection task | | |
| Beta band detection task | **Recognition index** |  |  |
|  | in focused attention only-tracking task | |  |
|  | in divided attention only-tracking task | |  |
|  | in focused attention detection task | | |
|  | in divided attention detection task | |  |
