# Supplementary material for: Attention and speech-processing related functional brain networks activated in a multi-speaker environment
Source: PLoS One. 2019 Feb 28;14(2):e0212754. doi: 10.1371/journal.pone.0212754 (PMC6394951; doi:10.1371/journal.pone.0212754)
Supplement: S4 Table — (DOCX) [file pone.0212754.s008.docx]

|  |  | ***t*** | **df** | ***p*** | **Cohen’s d** |
| --- | --- | --- | --- | --- | --- |
| **Delta** | Focused attention specific network | 10,522 | 24 | <0,001 | 0,854 |
|  | Divided attention specific network | -8,833 | 24 | <0,001 | 0,760 |
| **Low Alpha** | Focused attention specific network | 5,392 | 24 | <0,001 | 0,248 |
| **Beta** | Focused attention specific network | 7,005 | 24 | <0,001 | 0,605 |
|  | Divided attention specific network | -8,953 | 24 | <0,001 | 0,952 |
|  |  |  |  |  |  |
| **Delta** | Tracking task specific network | 7,355 | 24 | <0,001 | 0,957 |
| **Low Alpha** | Detection task specific network | -7,325 | 24 | <0,001 | 0,396 |
| **Beta** | Detection task specific network | -4,400 | 24 | <0,001 | 0,310 |
