# Supplementary material for: Attention and speech-processing related functional brain networks activated in a multi-speaker environment
Source: PLoS One. 2019 Feb 28;14(2):e0212754. doi: 10.1371/journal.pone.0212754 (PMC6394951; doi:10.1371/journal.pone.0212754)
Supplement: S5 Table — Each line represents a ROI (identified by its abbreviation as defined in S1 Table). ROIs are grouped by brain lobes (Frontal, Cingular, Temporal, and Parietal). The sum of connections within each lobe and the percentage of connections relative to all connections within subnetworks are also listed. (DOCX) [file pone.0212754.s009.docx]

|  | **Delta network** | | | | | | | **Low alpha network** | | | | | | **Beta network** | | | | | |
| --- | --- | --- | --- | --- | --- | --- | --- | --- | --- | --- | --- | --- | --- | --- | --- | --- | --- | --- | --- |
|  |  | **TASK TYPE** | | **ATTENTION** | | | | **TASK TYPE** | | **ATTENTION** | | | | **TASK TYPE** | | **ATTENTION** | | | |
|  |  | Tacking | | Focused | | Divided | | Detection | | Focused | | Divided | | Detection | | Focused | | Divided | |
|  |  | > | | > | | > | | > | | > | | > | | > | | > | | > | |
|  |  | Detection | | Divided | | Focosed | | Tracking | | Divided | | Focused | | Tracking | | Divided | | Focused | |
| **Frontal** |  | |  | |  | |  | |  | |  | |  | |  | |  | |  |
| SFG |  | | 1 | | 1 | | 3 | |  | |  | | 1 | | 2 | | 6 | |  |
| OFG lateral |  | |  | | 2 | |  | | 2 | | 4 | |  | | 1 | | 1 | |  |
| OFG medial |  | |  | | 3 | | 2 | | 2 | |  | |  | |  | | 2 | | 2 |
| MFG caudal |  | | 4 | | 5 | | 2 | |  | | 9 | |  | | 3 | | 2 | |  |
| MFG rostral |  | | 2 | | 6 | | 2 | | 4 | | 1 | |  | |  | | 2 | | 3 |
| IFG opercularis |  | | 1 | | 3 | |  | |  | | 2 | |  | |  | | 1 | | 4 |
| IFG orbitalis |  | |  | | 6 | | 1 | | 2 | |  | |  | | 1 | |  | | 2 |
| IFG triangularis |  | |  | | 3 | | 3 | |  | | 1 | |  | | 1 | | 2 | |  |
| PreCG |  | |  | | 2 | | 3 | |  | | 3 | |  | | 5 | | 2 | |  |
| **Sum** |  | | **8**  **(67%)** | | **31**  **(62%)** | | **16**  **(36%)** | | **10**  **(28%)** | | **20**  **(40%)** | | **1**  **(50%)** | | **13**  **(32%)** | | **18**  **(36%)** | | **11**  **(34%)** |
| **Cingular** |  | |  | |  | |  | |  | |  | |  | |  | |  | |  |
| ACG caudal |  | |  | | 1 | |  | |  | |  | |  | |  | | 5 | | 2 |
| ACG rostral |  | |  | | 1 | |  | |  | | 1 | |  | | 3 | | 2 | |  |
| PCG |  | |  | | 1 | | 2 | | 3 | |  | |  | | 4 | | 7 | | 1 |
| **Sum** |  | | **0** | | **3**  **(6%)** | | **2**  **(5%)** | | **3**  **(8%)** | | **1**  **(2%)** | | **0** | | **7**  **(18%)** | | **14**  **(28%)** | | **3**  **(9%)** |
| **Temporal** |  | |  | |  | |  | |  | |  | |  | |  | |  | |  |
| FFG |  | |  | | 1 | | 7 | | 2 | |  | |  | | 2 | | 1 | | 5 |
| ITG |  | |  | | 3 | | 2 | | 1 | | 7 | |  | | 5 | |  | | 2 |
| MTG |  | | 2 | | 2 | | 3 | | 3 | | 3 | |  | | 2 | | 4 | | 4 |
| STG |  | | 2 | | 2 | | 4 | | 4 | | 2 | |  | | 1 | |  | | 1 |
| HES |  | |  | | 1 | | 2 | | 3 | | 1 | | 1 | |  | |  | | 1 |
| **sum** |  | | **4**  **(33%)** | | **9**  **(18%)** | | **18 (41%)** | | **13**  **(36%)** | | **13**  **(26%)** | | **1**  **(50%)** | | **10**  **(25%)** | | **5**  **(10%)** | | **13**  **(41%)** |
| **Parietal** |  | |  | |  | |  | |  | |  | |  | |  | |  | |  |
| PreCUN |  | |  | | 1 | | 4 | | 2 | | 3 | |  | | 2 | | 3 | | 1 |
| SPG |  | |  | | 2 | |  | |  | | 2 | |  | | 2 | | 2 | | 2 |
| PoCG |  | |  | | 3 | |  | | 2 | | 3 | |  | | 3 | | 3 | |  |
| IPG |  | |  | |  | | 3 | | 1 | | 6 | |  | | 2 | |  | | 2 |
| SMG |  | |  | | 1 | | 1 | | 5 | | 2 | |  | | 1 | | 5 | |  |
| **Sum** |  | | **0** | | **7**  **(14%)** | | **8**  **(18%)** | | **10**  **(28%)** | | **16**  **(32%)** | | **0** | | **10**  **(25%)** | | **13**  **(26%)** | | **5**  **(16%)** |
