# Supplementary material for: Attention and speech-processing related functional brain networks activated in a multi-speaker environment
Source: PLoS One. 2019 Feb 28;14(2):e0212754. doi: 10.1371/journal.pone.0212754 (PMC6394951; doi:10.1371/journal.pone.0212754)
Supplement: S1 File — (DOCX) [file pone.0212754.s011.docx]

Two types of syntactic violations were generated, each with two subtypes: 1) subject-predicate agreement mismatch (M=12.2 SD=3.0, range: 6-19) with a plural subject noun and a singular predicate verb, where the subject could either precede or follow the predicate (Example: “A világháló kiépítésének kezdetei a huszadik század közepére nyúlik vissza.” (Hungarian original) “The origins [plural] of creating the worldwide web dates [singular] back to the mid-20th century.” (English translation)) and 2) subject-object reorganization errors (M=8.3, SD=3.1, range: 2-14) with either appending the object suffix to the subject word or removing the object suffix from the object word (Example: “A statisztikák szerint másodpercenként hetven új honlap hoznak létre világszerte.” (Hungarian original) “Statistics tell that people create seventy new web pages [object suffix missing] per second throughout the world.” (English translation). For the first type of syntactic violations, the violation is encountered at the point of hearing the mismatching second element of the intended agreement pair with the effect of recalculating the number of actors as subjects. Both variants of the second type of syntactic violations result in the reorganization of the syntactic structure of the sentence because when the listener encounters the intended subject with the object suffix appended or the intended object without the object suffix, they are incorrectly understood as the object or the subject, respectively. Then, at the point, where the syntactic violation is discovered (hearing the predicate verb), the listener needs to reassign the syntactic role of the affected word within the sentence. In a pilot study, Kocsis and colleagues (2017) found that, when the same sentences were presented visually one word at a time, all three types of syntactical violations elicited the Left Anterior Negativity (LAN) and/or the P600 component. The minimal distance between numerals and syntactic violations was 3 syllables.

Kocsis, Zs., Hajdu, B., Orosz, G., Winkler, I., & Honbolygó, F. (2017). Szintaktikai sértések és a sértés nyelvtani elemei közötti távolság hatásának vizsgálata a magyar nyelvben. *Magyar Pszichológiai Szemle, 72*(2), 149-162. doi:10.1556/0016.2017.72.2.1
