# Supplementary material for: Attention and speech-processing related functional brain networks activated in a multi-speaker environment
Source: PLoS One. 2019 Feb 28;14(2):e0212754. doi: 10.1371/journal.pone.0212754 (PMC6394951; doi:10.1371/journal.pone.0212754)
Supplement: S2 File — (DOCX) [file pone.0212754.s012.docx]

The localization error distance is defined as the Euclidean distance (mm) between the location of the maximum current distribution from the inverse solution (such as sLORETA) and the position of the true generating dipole (definition adapted from Song et al., 2015). Previous methodological studies that investigated the source reconstruction accuracy using simulated EEG source (i.e., “true dipoles”) found that the error distance mostly varied between 0.5. 2.0 cm (Plummer et al., 2008; Song et al., 2015; Huang et al., 2016; Pizzagalli., 2007; Baillet et al., 2001). Based on this we estimated for each cortical region the probability by which the reconstructed source activity could substantially overlap/influence with another cortical region’s source activity. In other words, we quantified how much of the ROI’s source signal could have been attributed to another source ROI region as a function of the localization error distance assumptions, testing the 1.5 - 2.0 cm range.

The assessment was performed with the following steps: 1) each volume grid of 15002 voxels in the inner head volume was assigned to its corresponding ROI; 2) Euclidian distance matrix was calculated by measuring the distance (cm) in the 3D space between all possible pairs of voxel’s centroids; 3) then the number of voxel distances belonging to a pair of ROIs were estimated that are below the threshold of 15mm and 20 mm separately. The 100 percent was defined as the squared sum of voxels that the smaller ROI consists of. 4) Matrixes with the spatial overlap percentages were created for all pairs of (62*62) cortical regions separately for 15, and 20mm threshold error distances. Matrix elements represent the percent of which two regions source reconstructed activity could be ambiguous due to localization error. 5) All ROIs pairs above the threshold degree of overlap (here set to 50 %, indicating that more than half of the smaller ROI’s voxel’s source activity could be unreliably attributed to another ROI. Ambiguous ROI pairs are plotted separately for 15 and 20 mm localization error value in S1 Fig, and listed in S2 Table.

The present EEG source localization solution with 15 mm estimated localization error could result in a high degree of overlap for 5 pairs of ROIs. These include mostly the smallest Heschl region from which the reconstructed source activity could be inaccurately from neighboring regions such as the supramarginal gyrus, insula, superior temporal postcentral (see S1 Fig; S2 Table). Source localization solution with 20 mm estimated localization error could affect 17 pairs of ROIs. Besides the Heschl region, other small volume ROIs source activity small volume could be ambiguously reconstructed. These regions are the entorhinal cortex, anterior cingulate cortices and the two subregions of the inferior frontal cortex (pars orbitalis and parstriangularis). Based on the results, only a few regions, those with the smallest volume could have been affected by mislocalization
