# Supplementary material for: Attention and speech-processing related functional brain networks activated in a multi-speaker environment
Source: PLoS One. 2019 Feb 28;14(2):e0212754. doi: 10.1371/journal.pone.0212754 (PMC6394951; doi:10.1371/journal.pone.0212754)
Supplement: S3 File — (DOCX) [file pone.0212754.s013.docx]

Connections exceeding the threshold with the F test statistic (TASK TYPE or ATTENTION contrast) are first collected in a set. The F values were computed for each connection between the EEG (N=946 edges [(44 × 43)/2) . The NBS algorithm then searched for largest (by size ; where size = number of edges) fully connected network from the set of selected nodes on each threshold level (3 ≥ F ≥ 10). Networks are defined as sets of nodes with each node consist at least a single supra-threshold connections. Permutation testing was used to set a family-wise error corrected p-value for each network. During permutation-based mass univariate testing, 10000 random networks were created by repeatedly permuting the assignment of condition and then selecting the largest networks at all F threshold level. The size (number of edges) of these networks from each permutation formed the distribution against which the family wise corrected networks were extracted. Networks with a size falling into the highest 5% of the distribution were regarded as significant. The significant networks obtained with the highest threshold within the F range (denoted by “K” when describing the statistical results) were selected for further network construction. Specifically, the final threshold was set by determining the maximum value that still resulted in at least one significant network for the given contrast, separately for the six EEG frequency bands. Next, within each network, the edges were ordered according to the size of the connectivity strength difference between the contrasted conditions, and the 50 edges with the highest difference were submitted to a post hoc pairwise t-test. Only edges with a significant (α=0.05) difference were selected for interpretation, as these characterize the largest FC strength difference for the contrast tested. Depending on the direction of the difference between the two contrasted conditions, the edges of the network identified by NBS were separated into two groups. For example, when testing the effects of attention, the edges that showed significantly higher FC strength in the focused relative to divided attention condition formed one group, whereas the ones showing the opposite effect, the other group.
