# Supplementary material for: Attention and speech-processing related functional brain networks activated in a multi-speaker environment
Source: PLoS One. 2019 Feb 28;14(2):e0212754. doi: 10.1371/journal.pone.0212754 (PMC6394951; doi:10.1371/journal.pone.0212754)
Supplement: S4 File — (DOCX) [file pone.0212754.s014.docx]

The effect size measure estimates a population parameter and thus it is not affected by the sample size. Consequently, effect size indicates the replicability of the statistical differences regardless of the number of samples that are included in the analysis. For this purpose, we computed the average connectivity strength for each identified network and condition. The average FC strength was calculated for the edges showing significantly stronger connectivity during the tracking than the detection task and vice versa (separately for the two task conditions) as well as for those showing significantly stronger FC during focused than divided attention and vice versa (separately for the two attention conditions). Pairwise dependent sample t-tests were then performed between the average network connectivity strength values and Cohen’s d effect sizes were calculated for measuring the standardized network strength differences for each contrast.
