# Supplementary material for: Attention and speech-processing related functional brain networks activated in a multi-speaker environment
Source: PLoS One. 2019 Feb 28;14(2):e0212754. doi: 10.1371/journal.pone.0212754 (PMC6394951; doi:10.1371/journal.pone.0212754)
Supplement: S5 File — (DOCX) [file pone.0212754.s015.docx]

NIRS signal was recorded simultaneously with the EEG using a 16-source/24- detector configuration (52 viable channels; see S1 Fig. for the channel distribution over the scalp) by a NIRStar 14.1 system (NIRx Medical Technologies, LLC). The sampling frequency was 7.81 Hz. The MNI coordinates of the channels were calculated using the 3D digitizer function (Singh, Okamoto, Dan, Jurcak & Dan, 2005) of the NIRS-SPM 4.1 software (Ye, Tak, Jang, Jung & Jang, 2009) from the configuration-based standard coordinates entered into the recording software (i.e., again, individual differences were neglected). Based on the MNI coordinates, the NIRS channels were spatially clustered into the 11 left and 11 right-hemispheric cortical regions (see S2 Fig; for list of the selected cortical regions with their abbreviations, see also S1 Table) by the xjView toolbox (http://www.alivelearn.net/xjview).

NIRS data were preprocessed by the NirsLab software (NIRx Medical Technologies, LLC). First, discontinuities indicating motion artifacts were corrected using NirsLAB’s built-in algorithm. Continuous data were then bandpass filtered (0.009-0.01Hz) and hemoglobin states (oxy- and deoxygenated hemoglobin concentration) were computed from the raw data using the modified Beer-Lambert Law. Preprocessed data for both oxy- and deoxygenated hemoglobin concentration changes were extracted for five-minute long intervals starting at the onset of the speech streams, separately for each stimulus block. The signals were then concatenated across stimulus blocks, separately for each participant and condition.

Singh, A. K., Okamoto, M., Dan, H., Jurcak, V., & Dan, I. (2005). Spatial registration of multichannel multi-subject fNIRS data to MNI space without MRI. *Neuroimage, 27*(4), 842-851. doi:10.1016/j.neuroimage.2005.05.019

Ye, J. C., Tak, S., Jang, K. E., Jung, J., & Jang, J. (2009). NIRS-SPM: statistical parametric mapping for near-infrared spectroscopy. *Neuroimage, 44*(2), 428-447. doi:10.1016/j.neuroimage.2008.08.036
