# Supplementary material for: Attention and speech-processing related functional brain networks activated in a multi-speaker environment
Source: PLoS One. 2019 Feb 28;14(2):e0212754. doi: 10.1371/journal.pone.0212754 (PMC6394951; doi:10.1371/journal.pone.0212754)
Supplement: S6 File — (DOCX) [file pone.0212754.s016.docx]

The strength of functional connectivity was quantified by using the absolute values of pairwise Pearson correlations for each pair of NIRS channels, yielding 52*52 adjacency FC matrices, separately for each participant and condition for the deoxygenated hemoglobin concentration. Only the deoxygenated NIRS signals were analyzed, because this signal best approximates the Blood-Oxygen-Level-Dependent (BOLD) response measured by fMRI and, therefore, it allows better comparison with future fMRI studies. In order to maximize the overlap between the EEG- and NIRS-based cortical regions, 22 regions of interest (ROI) were selected for further analysis (for the list of NIRS cortical regions see S1 Table). When multiple channels belonged to the same ROI, the value representing the functional connectivity strength of the ROI with another ROI was calculated by bootstrapping 1,000 times the connectivity values taken from all combinations of pairs of channels between the two ROIs and calculating the mean of the bootstrapped values, separately for each participant and condition. For ROIs represented by a single NIRS channel, the mean FC value between that channel and the channels of the other ROI were used. The procedure resulted in 22×22 dimension weighted adjacency FC matrices, separately for each participant and condition. The rest of the analysis and the visualization methods were identical to those described for the EEG FC analysis. Statistical analyisis of NIRS data was performed identically with EEG data statistics.
