# Supplementary material for: Attention and speech-processing related functional brain networks activated in a multi-speaker environment
Source: PLoS One. 2019 Feb 28;14(2):e0212754. doi: 10.1371/journal.pone.0212754 (PMC6394951; doi:10.1371/journal.pone.0212754)
Supplement: S7 File — (DOCX) [file pone.0212754.s017.docx]

Tracking task performance. Further, significant interaction was obtained between ATTENTION and LOCATION (F1,24=13.625; p=0.001 ηp2=0.362). Post-hoc pairwise comparisons showed higher recognition performance for the left than right target streams in the focused attention condition (p<0.001) and no significant difference in the divided attention condition. This was probably caused by the more salient prosodic style of the actor whose voice was delivered from the left loudspeaker, as his voice covered a higher dynamic range (~8.5 dB) compared to the other actor (~4.5 dB).
