# Supplementary material for: Attention and speech-processing related functional brain networks activated in a multi-speaker environment
Source: PLoS One. 2019 Feb 28;14(2):e0212754. doi: 10.1371/journal.pone.0212754 (PMC6394951; doi:10.1371/journal.pone.0212754)
Supplement: S8 File — (DOCX) [file pone.0212754.s018.docx]

For the NIRS deoxygenated hemoglobin concentration data a single functional network was identified which was affected by TASK TYPE (only tracking task vs. detection task). According to the post hoc analysis it had a large effect (t(24)=7,09 p<0,001;Cohen’s d=0,895). S6 Table shows the summary of the NIRS node degrees (the number of connections within subnetworks showing a significant ATTENTION or TASK TYPE effect), separately for each ROI. S3 Fig shows the subnetwork for NIRS deoxygenated hemoglobin concentration that was significantly affected by TASK TYPE (K=10.0; p < 0.001). The NIRS network comprises 28 edges connecting 17 nodes, all of which showed significantly (p<0.05, all) stronger connectivity during the tracking task relative to the detection tasks (S3 Fig). The subnetwork was found to be somewhat left lateralized, and the nodes with the highest degree (S6 Table) include frontal (MFG and SFG), temporal MTG, and parietal areas (mostly the SPG and SMG).
