# Supplementary material for: Attention and speech-processing related functional brain networks activated in a multi-speaker environment
Source: PLoS One. 2019 Feb 28;14(2):e0212754. doi: 10.1371/journal.pone.0212754 (PMC6394951; doi:10.1371/journal.pone.0212754)
Supplement: S9 File — (DOCX) [file pone.0212754.s019.docx]

The main effect of the DETECTION TASK TYPE on FC strength was tested by pairwise NBS based statistical contrasts which were performed separately for the six EEG frequency bands and the NIRS deoxygenated hemoglobin concentration: whether numeral and syntactic violation detection task FC networks are different - for this contrast, difference FC matrixes were calculated by separately subtracting the FC matrix obtained for the baseline condition from that obtained for the corresponding detection task condition FC matrices, separately for the two detection tasks but averaging across the divided attention conditions. For this test, the networks common to the detection tasks and the baseline have been removed, thus making the test more sensitive to the differences between the two detection tasks.

For none of the frequency bands, no significant subnetwork affected by DETECTION TASK TYPE was found even using the lowest threshold (F=3).
