# Supplementary material for: Attention and speech-processing related functional brain networks activated in a multi-speaker environment
Source: PLoS One. 2019 Feb 28;14(2):e0212754. doi: 10.1371/journal.pone.0212754 (PMC6394951; doi:10.1371/journal.pone.0212754)
Supplement: S10 File — (DOCX) [file pone.0212754.s020.docx]

The location of the task-relevant speech stream interacted with the attention factor in determining recognition performance, similarly for the two task conditions (see behavioral results). Therefore, post-hoc tests were conducted for assessing the effects of location and attention on significant subnetworks obtained through the NBS based procedure described above. For each edge of those networks, which were significantly affected by ATTENTION (focused vs. divided attention), a repeated measures ANOVA model was tested, separately for the six EEG bands and the NIRS deoxygenated hemoglobin concentration, using Matlab’s Statistics and Machine Learning Toolbox 10.1. In the model, ATTENTION was included as a within-subject and LOCATION (left vs. right detection task target stream) as a between-subject factor. The family-wise error was corrected using a permutation-based method. For each network, FC weights were permutated 1000 times randomly without replacement across conditions and participants, and the above described repeated measures ANOVA was tested on each permutation. For each permutation run, the highest F-values of the LOCATION main effect and of the ATTENTION × LOCATION interaction were separately registered. The p-value was established as the proportion of the previously extracted maximal F-values that were higher than or equal to the absolute value of the actual F-value, separately for the main effect and the interaction.

**Delta band:** Post-hoc ANOVAs revealed the following main effects of LOCATION on the ATTENTION-sensitive network (see S4 Table): three fronto-parietal connections (MFG-SPG; SFG- IPG; PreCG-PreCUN) were stronger when the speech stream that was the target of the tracking task was presented from the left rather than from the right side. Further a significant interaction between LOCATION and ATTENTION was found on two edges: between the left IFG and the right STG, which was stronger during focused than during divided attention, but only when the left speech stream was tracked; while the interhemispheric connectivity within the IFG cortices was stronger during focused than during divided attention, but only when the right speech stream was tracked.

**Alpha band:** Post hoc ANOVAs revealed only one edge with a significant interaction between LOCATION and ATTENTION (S4 Table): FC between the left MFG and PoCG was stronger during focused than during divided attention, but only when the left speech stream was tracked.

**Beta band:** Post-hoc ANOVAs revealed one connection within the ATTENTION-sensitive subnetwork on which there was a main effect of LOCATION (see a S3 Table): the connection between the right OFG and PCG was stronger when the right than when the left speech stream was tracked. Further, a significant interaction between LOCATION and ATTENTION was found on three edges: between the left ACC and right SMG and between the left PoCG and MFG, which were stronger during focused than during divided attention, but only when the right speech stream was tracked; while the connection between the right IPG and left HES was stronger during divided than during focused attention, but only when the right speech stream was tracked.
